# Mass Spectrometry Guided Discovery and Design of Novel Asperphenamate Analogs From *Penicillium astrolabium* Reveals an Extraordinary NRPS Flexibility

**DOI:** 10.3389/fmicb.2020.618730

**Published:** 2021-01-15

**Authors:** Karolina Subko, Xinhui Wang, Frederik H. Nielsen, Thomas Isbrandt, Charlotte H. Gotfredsen, Maria C. Ramos, Thomas Mackenzie, Francisca Vicente, Olga Genilloud, Jens C. Frisvad, Thomas O. Larsen

**Affiliations:** ^1^Department of Biotechnology and Biomedicine, Technical University of Denmark, Lyngby, Denmark; ^2^Department of Chemistry, Technical University of Denmark, Lyngby, Denmark; ^3^Fundación MEDINA, Granada, Spain

**Keywords:** natural product discovery, mass spectrometry, filamentous fungi, asperphenamate, amino acid incorporation, biological activity, NRPS flexibility

## Abstract

Asperphenamate is a small peptide natural product that has gained much interest due to its antitumor activity. In the recent years numerous bioactive synthetic asperphenamate analogs have been reported, whereas only a handful of natural analogs either of microbial or plant origin has been discovered. Herein we describe a UHPLC-HRMS/MS and amino acid supplement approach for discovery and design of novel asperphenamate analogs. Chemical analysis of *Penicillium astrolabium*, a prolific producer of asperphenamate, revealed three previously described and two novel asperphenamate analogs produced in significant amounts, suggesting a potential for biosynthesis of further asperphenamate analogs by varying the amino acid availability. Subsequent growth on proteogenic and non-proteogenic amino acid enriched media, revealed a series of novel asperphenamate analogs, including single or double amino acid exchange, as well as benzoic acid exchange for nicotinic acid, with the latter observed from a natural source for the first time. In total, 22 new asperphenamate analogs were characterized by HRMS/MS, with one additionally confirmed by isolation and NMR structure elucidation. This study indicates an extraordinary nonribosomal peptide synthetase (NRPS) flexibility based on substrate availability, and therefore the potential for manipulating and designing novel peptide natural products in filamentous fungi.

## Introduction

**FIGURE 1:**
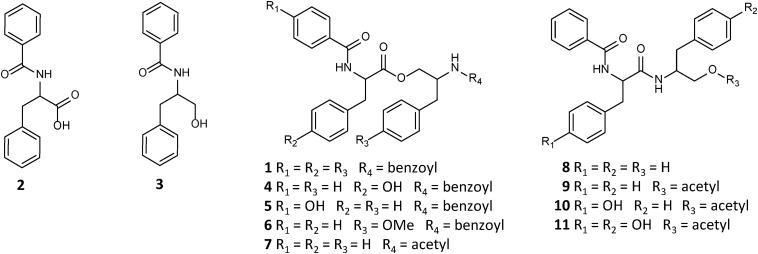
Structures of natural asperphenamate analogs.

Asperphenamate (**1**) is a linear amino acid (AA) ester, comprised of N-benzoylphenylalanine (**2**) and N-benzoylphenylalaninol (**3**). Asperphenamate, first discovered from *Aspergillus flavipes* in 1977 (Clark et al., 1977), was since found to be produced by a wide range of *Aspergillus* (Samson et al., 2011; Zheng et al., 2013; Ratnaweera et al., 2016; Hou et al., 2017) and *Penicillium* (Frisvad et al., 2004, 2013) species. Additionally, the compound has also been isolated in trace amounts from a number of unrelated plant species (Wu et al., 2004; Dang et al., 2014; Zhou et al., 2017; Bunteang et al., 2018; Caridade et al., 2018), suggesting endophytic fungi being the actual producers, rather than the plants. Although asperphenamate is mainly known for its antitumour activity and immense synthetic chemists interest in asperphenamate backbone modification (Li et al., 2012; Yuan et al., 2012, 2018, 2019, 2020; Liu et al., 2016), recent studies have also shown asperphenamate to be a potential neuroinflamatory inhibitor (Zhou et al., 2017), and to possess anti-HIV (Bunteang et al., 2018) and antidiabetic (Del Valle et al., 2016) properties. In recent years, a handful of new natural analogs have been isolated, namely Asperphenamates B (**4**) and C (**5**) (Liu et al., 2018), and 4-OMe-asperphenamate (Zheng et al., 2013; Ratnaweera et al., 2016) (**6**) from filamentous fungi. Other analogs containing partial structural similarities include: patriscabratine (**7**), a N-benzoylphenylalanine phenylalanynol acetate ester, aurantiamide (**8**) and aurantiamide acetate (**9**) (Zhou et al., 2017), N-benzoylphenylalanine phenylalanynol and phenylalanynol acetate amides, all isolated from plant material; cordyceamides A (**10**) and B (**11**) (Jia et al., 2009), a N-benzoyl-L-tyrosinyl-L-phenylalaninol and N-benzoyl-L-tyrosinyl-L-*p*-hydroxyphenylalaninol acetates, from an insect pathogen fungus (Figure 1); along with a number of tentatively identified related metabolites (Kildgaard et al., 2014; Sica et al., 2016).

Biosynthesis of asperphenamate was first described in the filamentous fungus, *P. brevicompactum* (Li et al., 2018). Here, a two module NRPS system was described, where the first module, ApmA, is responsible for amide bond catalysis between the phenylalanine and benzoic acid moieties, and subsequent reduction to afford N-benzoylphenylalaninol (**3**), while the second module, ApmB, utilizes the same substrates to produce N-benzoylphenylalanine (**2**), as well as catalyses the ester bond formation between the two intermediates to release the final product asperphenamate (**1**). Assuming, that other filamentous fungi may follow the same or a similar biosynthetic pattern, the production of **4–5** in *Penicillium sp.* and **6** *in Aspergillus sp.*, involving tyrosine, 4-OMe-phenylalanine and 4-hydroxybenzoic acid instead of phenylalanine and benzoic acid as substrate molecules, indicates promiscuity of either one or both NRPS modules and provides new insights for production of novel asperphenamate analogs and lays the grounds for molecular biology work to achieve higher production of asperphenamate and related analogs.

To contribute to a better understanding of the diversity of asperphenamate biosynthesis and address the increasing resistance toward anticancer drugs (Vasan et al., 2019), *P. astrolabium* IBT 28865, a distant relative of *P. brevicompactum* from section *Brevicompacta* (Serra and Peterson, 2007), was investigated for production of asperphenamate and related analogs. In this study, we employed an ultra-high performance liquid chromatography diode array detection quadrupole time of flight high-resolution tandem mass spectrometry (UHPLC-DAD-QTOF-HRMS/MS) to dereplicate known and novel asperphenamate analogs. As a result, 22 novel asperphenamate analogs were characterized by HRMS/MS, of which 21 were designed using proteogenic and non-proteogenic AAs as a supplement to the growth media. This study has further revealed a rare promiscuity of a fungal NRPS, laying the grounds for future NRPS research in filamentous fungi. Altogether, this study demonstrates the HRMS/MS based dereplication and characterization of novel analogs of a known bioactive peptide scaffold to be a powerful strategy in natural product discovery.

## Materials and Methods

### Reagents, Strains, and Media

All solvents and reagents were purchased from Sigma-Aldrich (St. Louis, MO, United States), for the exception of *para*-substituted phenylalanines, which were acquired from Bachema (Bubendorf, Switzerland); ultra-pure water used throughout the study was filtered with a Milli-Q system (Millipore, Burlington, MA, United States).

*Penicillium astrolabium* (IBT 28865), *P. olsonii* (IBT 28864), *P. bialowiezense* (IBT 28294), and *P. brevicompactum* (IBT 30524) are filamentous fungi from the IBT culture collection at the Department of Biotechnology and Biomedicine, Technical University of Denmark.

For the chemical profile analysis, *P. astrolabium* was cultivated with 3-point inoculation on Czapek yeast agar (CYA), yeast extract sucrose agar (YES) and malt extract agar (MEA; Oxoid) for 7, 10, and 14 days at 20 and 25°C in the dark. For large scale cultivation, the fungus was cultivated with 3-point inoculation on 200 YES agar plates, and incubated for 10 days at 25°C in the dark. For a proteogenic AA incorporation study, the fungus was cultivated with 3-point inoculation on Czapek (CZ) agar plates (10 cm) for 14 days at 25°C in the dark. Here, triplicates of 24 sets of supplemented media were used: 20 with all proteogenic AAs at 5 g/L; two for anthranilic acid and 4-hydroxybenzoic acid at 2.5 g/L, and two with additional inorganic nitrogen supplement of NaNO_3_ at 5 g/L and 10 g/L. For a non-proteogenic AA incorporation study, the fungus was cultivated with 1-point inoculation on CZ agar plates (6 cm) for 14 days at 25°C in the dark. Here, triplicates of four sets of 4-chloro-L-phenylalanine, 4-bromo-L-phenylalanine, 4-amino-L-phenylalanine, and 4-nitro-L-phenylalanine supplemented media were used at 2.5 g/L.

For the chemical profile analysis and comparison, all four fungi were cultivated with 3-point inoculation on minimal media (MM), CZ, CYA, and YES 10 cm agar plates for 7 days 25°C in the dark.

### Extraction and Isolation

For chemical profiling and the asperphenamate analog design study, five 6 mm diameter plugs were taken in triplicates and extracted with acidic (1% formic acid; FA) isopropanol (iPr) – ethyl acetate (EtOAc) (1:3 v/v) as described by Smedsgaard (1997). All samples were re-dissolved ultrasonically for 10 min in 100 μL methanol (MeOH) and centrifuged prior to analysis by LC-MS.

For large-scale extraction, the agar plates were extracted twice with acidic (1% FA) EtOAc. The liquid-liquid partitioning was then performed on the crude extract, by dissolving it with 90% MeOH:water and treating it with the same amounts of heptane, resulting in two phases. After separating the heptane phase, the 90% MeOH:water fraction was then diluted with water to get 50% MeOH:water, and further treated with dichloromethane (DCM), resulting in three phases overall. The DCM phase was dried before loading onto a 50 g SNAP column (Biotage, Uppsala, Sweden) with diol material (Isolute diol, Biotage). Crude fractionation was performed using an Isolera One automated flash system (Biotage) with stepwise increments of 25% at 50 mL/min in heptane-DCM-EtOAc-MeOH system, starting at 100% heptane, finishing at 100% MeOH, resulting in 13 fractions (i.e., heptane, heptane 3:1 DCM, heptane:DCM, heptane 1:3 DCM, DCM, DCM 3:1 EtOAc, DCM:EtOAc, DCM 1:3 EtOAc, EtOAc, EtOAc 3:1 MeOH, EtOAc:MeOH, EtOAc 1:3 MeOH, and MeOH), with 300 mL each. Selected resulting fractions were further fractionated on a 25 g SNAP column with RP C18 material (Grace, 15 μm/100 Å) at a flow rate of 30 mL/min using a stepwise 30–100% MeOH:water (both buffered with 50 ppm TFA) gradient as follows: in 10% increments at 30–50, 5% increments of 50–80, and 10% increments of 80–100%, resulting in 11 fractions (i.e., 30, 40, 50, 55, 60, 65, 70, 75, 80, 90, and 100%). Further separation was achieved on an Agilent Infinity 1290 HPLC-DAD (Agilent Technologies, Santa Clara, CA, United States) system, with UV monitoring at 230 and 280 nm, a flow rate of 4 mL/min and column temperature at 40°C as follows: Asperphenamate (**1**) and Asperphenamate L (**13**) were purified on a Gemini C_6_-Phenyl column (5 μm, 110 Å, 250 × 10 mm, Phenomenex) using a linear gradient of 57 to 64% acetonitrile (MeCN)/water over 30 min; Asperphenamate W (**12**) on a Kinetex RP C18 column (5 μm, 100 Å, 250 × 10 mm, Phenomenex) using a linear gradient of 55 to 65% MeCN/water over 20 min at a flow rate of 4 mL/min; Asperphenamate Y (**4**) and Asperphenidine F1 (**1a**) on a Kinetex RP C18 column (5 μm, 100 Å, 250 mm × 10 mm, Phenomenex) using a linear gradient of 55 to 67% MeOH/water over 20 min at a flow rate of 4 mL/min. All solvents were buffered with 50 ppm TFA.

*Asperphenamate* (**1**): white powder; [α]^20^_D_ −25.5° (c 0.11, CHCl_3_); UV (MeCN) λ_max_ 238 and 272 sh nm; ^1^H and ^13^C NMR data, see Table 1 and Supplementary Figure S1; HRESIMS m/z 507.2279 [M+H]^+^ (calculated for C_32_H_30_N_2_O_4_, m/z 507.2278).

**TABLE 1:** ^1^H and ^13^C NMR shifts for asperphenamates F (1), Y (4), and W (12) and L (13) in chloroform (CDCl_3_).

	1		4		12		13	
Position	δ_C_*	δ_H_ (*J* in Hz)	δ_C_	δ_H_ (*J* in Hz)	δ_C_	δ_H_ (*J* in Hz)	δ_C_	δ_H_ (*J* in Hz)
1			167.7		167.6		167.8	
2			133.4		133.5		133.3	
3	127.0	7.70 dd (8.3, 1.1)	127.2	7.65 m	127.3	7.63 m	127.1	7.72 m
4	128.6	7.39 m	128.8	7.39 m	128.6	7.29 m	128.6	7.41 m
5	131.9	7.50 tt (7.5, 1.1)	132.2	7.50 tt (7.4, 1.2)	132.1	7.48 t (7.4)	132.2	7.52 m
1′			172.2		172.5		173.2	
2′	54.4	4.92, q (6.6)	54.8	4.87 d (6.7)	54.3	5.04 q (6.5)	52.1	4.71 m
3′ NH		6.58 d (6.6)		6.59 d (6.6)		6.69 d (6.4)		6.46 d (6.8)
4′	37.5	3.29 dd (14.0, 6.6)	36.9	3.20 dd (14.0, 6.5)	27.7	3.43 d (6.0)	40.8	1.79 m
		3.21 dd (14.0, 7.0)		3.14 dd (14.0, 6.9)				1.69 m
5′			127.6		110.1		25.1	1.75 m
6′	129.1	7.21 m	130.5	7.05 m	127.5		22.2	0.99 d (6.5)
7′	128.8	7.29 m	116	6.76 m	118.7	7.64 m	22.8	1.02 d (6.5)
8′	126.7	7.24 m	155.3		120.1	7.12 t (7.4)		
9′					122.7	7.20 m		
10′					111.6	7.33 d (7.9)		
11′					136.4			
12′ NH						8.06 s		
13′					123.1	7.06 d (2.2)		
1″	65.3	4.54 dd (11.4, 3.4)	65.5	4.50 dd (11.4, 3.6)	65.4	4.46 dd (11.6, 3.6)	65.1	4.59 dd (11.5, 3.3)
		4.04 dd (11.4, 4.4)		4.04 dd (11.4, 4.5)		4.06 dd (11.6, 4.6)		4.08 dd (11.5, 4.6)
2″	50.2	4.62 m	50.6	4.60 m	50.6	4.55 m	50.5	4.69 m
3″ NH		6.67 d (8.4)		6.71 d (8.4)		6.59 d (8.4)		6.73 d (8.2)
4″	37.2	3.00 dd (13.7, 6.4)	37.4	3.01 dd (13.9, 6.5)	37.3	2.94 dd (13.6, 6.7)	37.3	3.10 dd (13.6, 6.5)
		2.89 dd (13.8, 8.5)		2.91 dd (13.9, 8.2)		2.81 dd (13.6, 8.4)		3.01 dd (13.8, 8.2)
5″			137.1		137.4		137.3	
6″	129.2	7.23 m	129.5	7.22 m	129.5	7.18 s (7.6)	129.3	7.29 m
7″	128.3	7.32 m	128.9	7.31 m	128.7	7.36 t (7.8)	128.7	7.32 m
8″′	127.3	7.25 m	127.0	7.24 m	126.9	7.23 m	126.8	7.25 m
1″′			167.8		167.4		167.3	
2″′			134.1		134.4		134.3	
3″′	126.9	7.65 dd (8.3, 1.1)	127.3	7.68 m	127.2	7.63 m	127.1	7.70 m
4″	128.6	7.31 m	128.6	7.31 m	128.8	7.29 m	128.4	7.28 m
5″′	131.3	7.43 tt (7.5, 1.1)	131.7	7.43 tt (7.4, 1.1)	131.5	7.43 t (7.4)	131.3	7.42 m

*^13^C NMR data available only from HSQC experiment.

*Asperphenamate Y* (**4**): white powder; [α]^20^_D_ −25.8° (c 0.12, CHCl_3_); UV (MeCN) λ_max_ 239 and 275 sh nm; ^1^H and ^13^C NMR data, see Table 1 and Supplementary Figure S2; HRESIMS m/z 523.2227 [M+H]^+^ (calculated for C_32_H_30_N_2_O_5_, m/z 523.2227).

*Asperphenamate W* (**12**): white powder; [α]^20^_D_ −43.6° (c 0.14, CHCl_3_); UV (MeCN) λ_max_ 236 and 278 nm; ^1^H and ^13^C NMR data, see Table 1 and Supplementary Figure S3; HRESIMS m/z 546.2389 [M+H]^+^ (calculated for C_34_H_31_N_3_O_4_, m/z 546.2387).

*Asperphenamate L* (**13**): white powder; UV (MeCN) λ_max_ 237 and 272 sh nm; ^1^H and ^13^C NMR data, see Table 1 and Supplementary Figure S4; HRESIMS m/z 473.2436 [M+H]^+^ (calculated for C_29_H_32_N_2_O_4_, m/z 473.2435).

*Asperphenidine F1* (**1a**): white powder; UV (MeCN) λ_max_ 238 and 272sh nm; ^1^H NMR data, see Supplementary Table S5 and Supplementary Figure S5; HRESIMS m/z 508.2230 [M+H]^+^ (calculated for C_3__1_H_29_N_3_O_4_, m/z 508.2231).

### UHPLC-DAD-QTOF-MS Analysis

All samples were analyzed on an Agilent Infinity 1290 UHPLC system (Agilent Technologies, Santa Clara, CA, United States) equipped with a diode array detector (DAD), monitoring between 190 and 640 nm. Separation was achieved on an Agilent Poroshell 120 phenyl-hexyl column (150 mm × 2.1 mm, 1.9 μm particles) with a flow rate of 0.35 mL/min at 40°C, using a linear acetonitrile (MeCN)/water (both buffered with 20 mM FA) gradient of 10 to 100% MeCN in 10 min, followed by 2 min flush at 100% MeCN, return to starting conditions in 0.1 min and equilibration at 10% for 2 min before the following run. It was coupled to an Agilent 6545 QTOF MS equipped with Dual Jet Stream ESI source with the drying gas temperature of 250°C and gas flow of 8 L/min and sheath gas temperature of 300°C and flow of 12 L/min, capillary voltage 4,000 V and nozzle voltage of 500 V. The mass spectrometer was operated in positive polarity, recording centroid data in m/z range 100 to 1,700 for MS mode, and 30–1,700 for MS/MS mode, with acquisition rate of 10 spectra/s. Automated HRMS/MS was done for ions detected in the full scan above 50,000 counts, with a cycle time of 0.5 s, quadrupole width of m/z ± 0.65 using fixed CID energies of 10, 20, and 40 eV with maximum three precursor ions per cycle. A lock mass solution of 70% MeOH was infused in the second sprayer, with an extra LC pump at a flow of 15 μL/min using a 1:100 splitter. The solution contained 1 μM tributylamine (Sigma-Aldrich) and 10 μM hexakis(2,2,3,3-tetrafluoropropoxy)phosphazene (Apollo Scientific Ltd., Cheshire, United Kingdom) as lock masses. The [M + H]^+^ ions of both compounds (m/z 186.2216 and 922.0098, respectively) were used.

In-house fungal metabolite library search was done as described by Kildgaard et al. (2014). Data files were processed in MassHunter workstation B.07.00 with “Find by Auto MS/MS function” with a processing limit to 200 largest peaks and mass match tolerance m/z 0.05. HRMS/MS library search was performed using parent and fragment ion accuracy of 20 ppm + 2 mDa, with minimal forward score of 50 and reverse score of 80.

Targeted analysis for the asperphenamate analog design study was performed using expected masses of individual AA and benzoic acid analogs, for all potential precursors, intermediates and final products, see Supplementary Table S1. Relative amounts of asperphenamate analogs were quantified by direct integration of peak area of target compounds, normalized to xanthoepocin water loss adduct peak area ([M-H_2_O+H]^+^ m/z 589.0975) in control samples. All analyses were performed in triplicates. All MS/MS spectra reported were at 20 eV, unless stated otherwise.

### General Experimental Procedures

1D and 2D NMR analyses were performed on a Bruker Avance 800 MHz spectrometer (Bruker, Billerica, MA, United States), using standard sequence pulses. Samples were analyzed in a 3 mm TCl cryoprobe using deuterated chloroform (CDCl_3_) and referenced to the residual solvent signals δ_H_ = 7.26 ppm and δ_C_ = 77.16 ppm. J-couplings are reported in hertz (Hz) and chemical shifts (δ) in ppm. For 1D and 2D NMR data, see Supplementary Tables 2–4 and Supplementary Figures 1–5.

Optical rotations were measured in chloroform (CHCl_3_) on a PerkinElmer 341 Polarimeter (PerkinElmer, Waltham, MA, United States).

### Cytotoxicity Assay

Compounds **1**, **1a**, **4**, **12**, and **13** were tested in triplicates against five cancer cell lines, i.e., human lung carcinoma A549 ATCC CCL-185, breast adenocarcinoma MCF7 ATCC HTB-22, human skin melanoma A2058 ATCC CRL-11147, hepatocyte carcinoma HepG2 ATCC HB-8065 and pancreas carcinoma MiaPaca-2 ATCC CRL-1420 following previously described methodology (Audoin et al., 2013; Lauritano et al., 2020).

## Results

### Chemical Profile of *Penicillium astrolabium*

**FIGURE 2:**
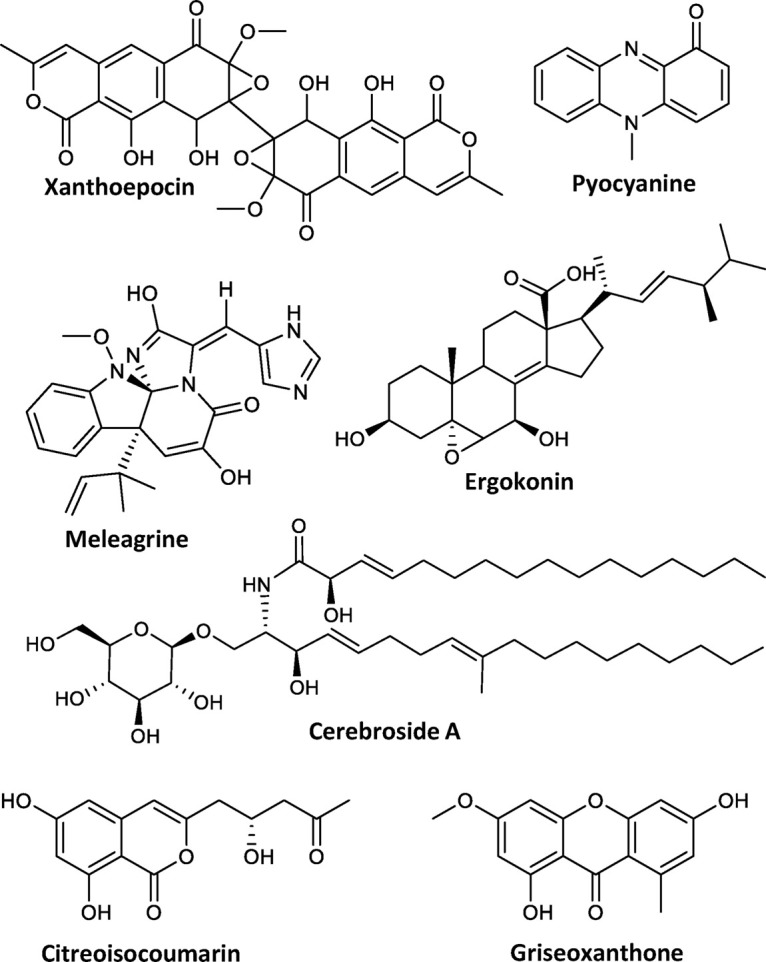
Compounds produced by *P. astrolabium* based on the in-house database search.

To investigate the secondary metabolite profile from *P. astrolabium*, the fungus was inoculated on three media (MEA, CYA, and YES) and incubated at 20 and 25°C for 7, 10, and 14 days. The resulting 18 extracts were analyzed by UHPLC-DAD-QTOF-MS and used for automated in-house library search of fungal secondary metabolites (Kildgaard et al., 2014). In addition to previously reported asperphenamate (**1**), N-benzoylphenylalanine (**2**) and xanthoepocin (Perrone et al., 2015), all 18 extracts also revealed the presence of meleagrin and its biosynthetic intermediates neoxaline, glandicoline B, roquefortine C and histidyltryptophanyldiketopiperazine (Ali et al., 2013), as well as cerebroside A. Other notable secondary metabolites include ergokonin B and pyocyanine detected on YES and CYA media extracts; citreoisocoumarin detected only in YES extracts; griseoxanthone C mainly seen on CYA 20°C (Figure 2). Additionally, a series of di- and tetracyclopeptides with varying AA composition, depending on growth conditions, were produced.

### Targetted Asperphenamate Daughter Ion Search Reveals Novel Analogs

**FIGURE 3:**
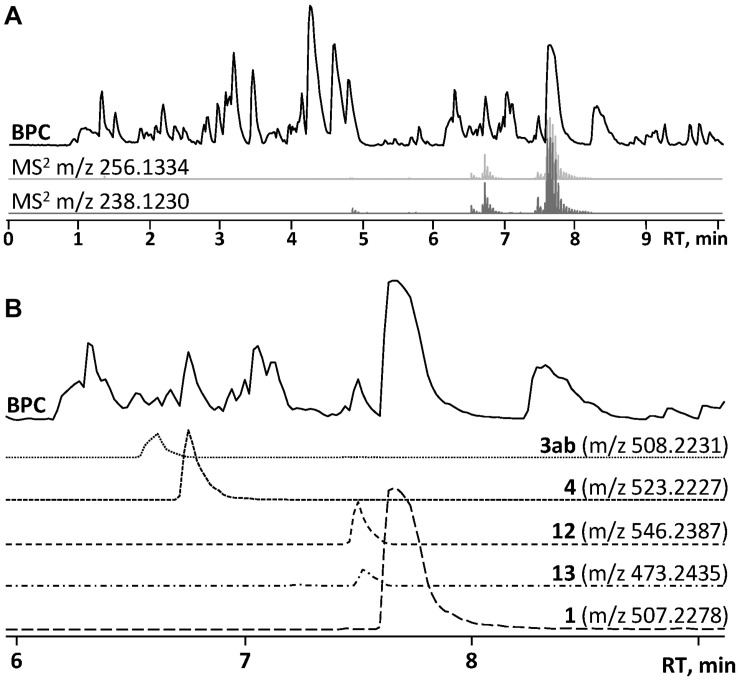
HPLC-MS analysis for asperphenamate analog production on YES media. **(A)** Base peak chromatogram (BPC) of crude *P. astrolabium* extract, with extracted ion chromatogram (EIC) showing “bait” ions m/z 256.1334 and m/z 238.1230. **(B)** Zoomed BPC with EIC for asperphenamate analogs at full scan.

**FIGURE 4:**
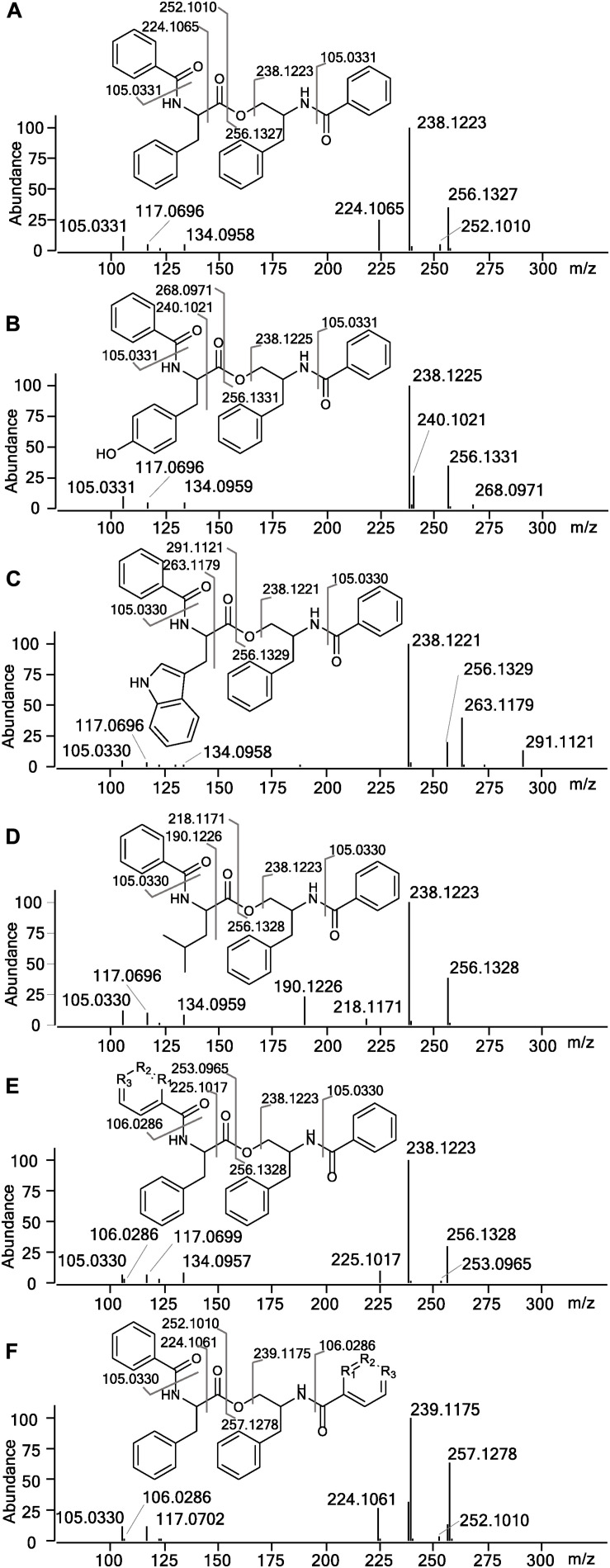
MS/MS spectra and assignment of asperphenamate analogs. **(A)** Asperphenamate m/z 507.2231, **(B)** tyrosine analog m/z 523.2227, **(C)** tryptophan analog m/z 546.2387, **(D)** leucine analog m/z 473.2435, and **(E,F)** pyridinecarboxilic acid analogs, where R_1_, R_2_, or R_3_ = N.

In the HRMS/MS analysis, asperphenamate (**1**) and reported fungal analogs (**4–6**) share two major fragment ions m/z 256.1334 and m/z 238.1230, corresponding to ester bond cleavage to result in a N-benzoylphenylalaninol protonated ion [C_16_H_18_NO_2_]^+^, followed by water loss on the same moiety to get [C_16_H_16_NO]^+^. In addition, a minor fragment of m/z 105.0335 [C_7_H_5_O]^+^ corresponding to a benzoyl loss was also observed. To screen for potential asperphenamate analogs, the two major fragment ions were used as “bait” (Figure 3A), resulting in five major peaks (Figure 3B): asperphenamate ([M+H]^+^ m/z 507.2277, C_32_H_30_N_2_O_4_), the most abundant analog with an extra oxygen atom ([M+H]^+^ m/z 523.2227, C_32_H_30_N_2_O_5_), an analog indicating a single carbon-nitrogen exchange ([M+H]^+^ m/z 508.2232, C_31_H_29_N_3_O_4_), and two other analogs with a significant mass differences, one 34Da lower ([M+H]^+^ m/z 473.2436, C_29_H_32_N_2_O_4_) and the other 39Da higher ([M+H]^+^ m/z 546.2385, C_34_H_31_N_3_O_4_) to that of asperphenamate. Subsequent MS/MS analysis revealed, that two asperphenamate-specific fragments m/z 252.1010 and m/z 224.1064, corresponding to ester cleavage and subsequent CO loss of an N-benzoylphenylalanine moiety (Figure 4A), have been exchanged for fragments 16Da higher, namely m/z 268.0971 and m/z 240.1021 (Figure 4B), for compound with m/z 523.2227. Therefore, with the fragment of m/z 105.0331 being present in both of compounds, and no other major differences in fragmentation patterns observed, a phenylalanine exchange for tyrosine in a non-reduced N-benzoyl AA moiety could be proposed, resulting in **4**. The asperphenamate analog with m/z 546.2385 produced unique fragments of m/z 291.1121 [C_18_H_15_N_2_O_2_]^+^ and m/z 263.1179 [C_17_H_15_N_2_O]^+^ (Figure 4C). Taking into account the presence of a benzoyl ion [C_7_H_5_O]^+^, the rest of m/z 291.1121 fragment suggest molecular formula C_11_H_10_N_2_O, corresponding to phenylalanine exchange for tryptophan in a non-reduced N-benzoyl AA moiety. Similarly, the differences between the unique fragments in m/z 473.2436 and the benzoyl ion led to proposal of leucine containing analog (Figure 4D). Finally, a compound with m/z 508.2232 showed similar fragmentation patterns to those of asperphenamate, however, fragments corresponding to fragmentation of a non-reduced AA moiety showed a fragment mass increase by 1Da, with a fragment ion m/z 106.0286 [C_6_H_4_NO]^+^ indicating a pyridinecarboxylic acid incorporation (Figure 4E). Moreover, MS/MS data revealed trace amounts of a coeluting isomer, with the two major ion fragments weighing 1 Da higher, namely m/z 257.1278 and m/z 239.1175, hence indicating that the pyridinecarboxylic acid can also be incorporated into the reduced AA part of the molecule (Figure 4F).

### NMR Confirms Phenylalanine Exchange for Other Amino Acids in the Non-reduced N-Benzoyl Amino Acid Moiety

**FIGURE 5:**
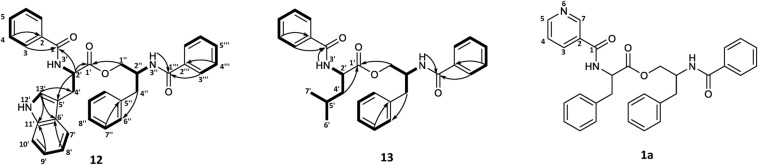
COSY and key HMBC correlations for asperphenamates W (**12**) and L (**13**) and proposed structure for asperphenidine F1 (**1a**).

To confirm the structures proposed by HRMS/MS fragmentation patterns, a large scale of 200 agar plates was grown for targeted isolation of asperphenamate (**1**), and tyrosine (**4**), tryptophan (**12**) and leucine (**13**) analogs, as well as one of the pyridinecarboxylic acid analogs (**1a**). ^1^H and ^13^C NMR data shown in Table 1, with full assignment table and spectra available in supplementary material (Supplementary Tables 2–4 and Supplementary Figures 1–5). Data for asperphenamate (**1**) and the tyrosine analog (**4**) fit with previously published data (Catalán et al., 2003; Liu et al., 2018). ^1^H and ^13^C NMR shifts of N-benzoylphenylalaninol and the N-benzoyl part of non-reduced AA moiety was in agreement within all four compounds, further supported by COSY and HMBC correlations for tryptophan (**12**) and leucine (**13**) analogs (Figure 5 and Supplementary Tables 3–4).

The rest of the shifts corresponding to **12** showed three spin systems, with the first comprised of an amino group at NH-3′ (δ_H_ 6.69), a methine at H-2′ (δ_H_ 5.04) and a methylene at H-4′ (δ_H_ 3.43), the second consisted of four aromatic methines at H-7′ (δ_H_ 7.64), H-8′ (δ_H_ 7.12), H-9′ (δ_H_ 7.20) and H-10′ (δ_H_ 7.33), and the third one included aromatic amino and methine groups, NH-12′ (δ_H_ 8.06) and H-13′ (δ_H_ 7.06), respectively. The HMBC correlations of the last two spin systems from H-7′ and H-13′ to C-5′ (δ_C_ 110.1), H-8′ and H-13′ to C-6′ (δ_C_ 127.5), and H-9′ and H-13′ to C-11′ (δ_C_ 136.4), revealed the presence of indole, which was connected to the first spin system by H-2′ to C-5′ (δ_C_ 110.1) and H-4′ to C-13′ (δ_C_ 123.1), to confirm presence of tryptophan. The HMBC correlations from H-3 (δ_H_ 7.63) and H-2′ to C-1 (δ_C_ 167.6) and H-2′ and H-1″ (δ_H_ 4.46/4.06) to C-1′ (δ_C_ 172.5), connected tryptophan moiety to the benzoyl and N-benzoylphenylalaninol parts of the molecule (Figure 5).

For **13**, the rest of the shifts comprised a single spin system of amino group NH-3′ (δ_H_ 6.46), two methines at H-2′(δ_H_ 4.71) and H-5′(δ_H_ 1.75), a diastereotopic methylene at H-4′(δ_H_ 1.79/1.69), and two methyl groups at H-6′(δ_H_ 0.99) and H-7′(δ_H_ 1.02), to give a leucine backbone. The spin system was connected to the rest of the structure by H-3 (δ_H_ 7.72) and H-2′ to C-1 (δ_C_ 167.8) and H-2′ and H-1′ (δ_H_ 4.59/4.08) to C-1′ (δ_C_ 173.2) (Figure 5). The NMR data was eventually found to be in agreement with the commonly overlooked lichen secondary metabolite hypothallin (Huneck et al., 1992).

Compound **1a** was purified in trace amounts (0.2 mg) and only 1H NMR was acquired (Supplementary Figure S5). In comparison to asperphenamate, all the proton shifts in the aliphatic range for **3a** were of same multiplicity and similar shift values, as well as shifts for both amino groups. In the aromatic range, three asperphenamate shifts at H-3 (δ_H_ 7.70), H-4 (δ_H_ 7.39) and H-5 (δ_H_ 7.50) were swapped for more downfield shifts at δ_H_ 7.94 (m), δ_H_ 8.72 (dd), and δ_H_ 8.87 (d). Based on the two latter shifts and their multiplets, they were assigned as H-5 and H-7, respectively, with δ_H_ 7.94 (m) assigned at H-3, and the H-4 shift assigned to the general aromatic region at 7.18–7.35, led to confirmation of pyridinecarboxylic acid moiety as nicotinic acid. This fits with the published NMR data for nicotinic acid (Chen et al., 1999) and the corresponding synthetic asperphenamate analog (Liu et al., 2016).

Herein we propose a new asperphenamate analog naming system using one letter AA abbreviation to denote a specific AA incorporation, based on similar azaphilone pigment naming system proposed by Isbrandt et al. (2020). Compounds **12** was named asperphenamate W, whereas compounds **4** and **13** will be referred to as asperphenamate Y and L, respectively. Compound **1a** was named asperphenidine F1, to signify it being an asperphenamate analog, with phenylalanine incorporation and benzoic acid exchange for nicotinic acid at the non-reduced part of the molecule.

### Amino Acid Enriched Media Induces Phenylalanine Exchange in the N-Benzoylphenylalanine Moiety

**FIGURE 6:**
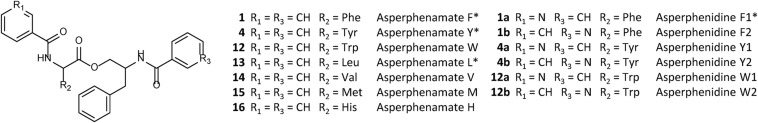
Structural overview and proposed naming system of HRMS/MS characterized asperphenamate analogs from *P. astrolabium* upon proteogenic amino acid supplement study. Structures marked with asterisk indicate previously reported compounds either of natural or synthetic origin.

To investigate if higher AA availability can induce AA exchange in asperphenamate biosynthesis, the fungus was incubated on CZ media supplemented with one of each of the 20 proteogenic AAs. Subsequently, targeted MS analysis was performed by search of masses corresponding to phenylalanine exchange for one AA moiety within the asperphenamate backbone (Supplementary Table S1). In addition tyrosine (**4**), tryptophan (**12**), and leucine (**13**) analogs, the novel valine (**14**), methionine (**15**), histidine (**16**), alanine and isoleucine analogs could also be observed, and AA exchange in the non-reduced AA part of the molecule was confirmed for compounds **14–16** by HRMS/MS fragmentation patterns (Figure 6 and Supplementary Figure S6). The new analogs were accordingly named as asperphenamates V, M, and H.

**FIGURE 7:**
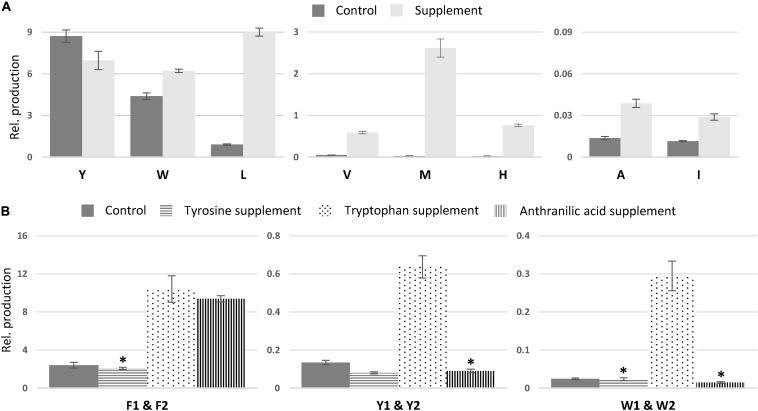
Relative production of asperphenamate and asperphenidine analogs. **(A)** Relative production of asperphenamate amino acid analogs in control and corresponding amino acid supplement media. **(B)** Production of asperphenidines F, Y, and W in control, tyrosine, tryptophan and anthranilic acid supplemented media. Error bars show standard deviation. All data, except the ones marked with asterisk, indicate statistically significant differences compared to the control (*p* < 0.05).

In comparison to non-fed control cultures of previously characterized compounds, only small changes in production were observed for asperphenamate Y and W production, a slight decrease and increase, respectively (Figure 7A). However, upon leucine supplement, asperphenamate L production increased 10-fold in comparison to the control. A similar increase pattern was also observed in the valine supplement experiment, whereas the production of histidine and methionine analogs was drastically boosted upon respective AA supplement, with 33-fold increase for asperphenamate H and more than a 100-fold increase for asperphenamate M. Although, the production of alanine and isoleucine analogs increased by three-fold for each, the relative amounts were still marginally lower in comparison to other uptake experiments, and were not sufficient for MS/MS data acquisition and assignment of structures (Figure 7A).

Additionally, MS analysis targeting reduced and non-reduced AA N-benzoyl precursors, and single or double AA exchange in asperphenamate, patriscabratine, aurantiamide and aurantiamide acetate backbones, was performed to result in the discovery of an additional double leucine asperphenamate analog characterized by HRMS/MS (Supplementary Figure S6). No other AA analogs or analogs for benzoic acid exchange for 4-hydroxybenzoic or anthralinic acids were observed.

### Tryptophan Induces Nicotinic Acid Incorporation

In contrast to the expected asperphenamate W (**12**) being one of the major metabolites upon growth on tryptophan supplemented media, both asperphenidines F1 and F2 (**1ab**), with nicotinic acid exchange on either the non-reduced or reduced part of the molecule, respectively, showed the most drastic increase in the relative amount in comparison to the control. With the similar behavior observed in anthranilic acid supplement, targeted MS analysis of all extracts was performed with masses corresponding to the benzoic acid exchange for nicotinic acid for novel AA analogs described above (Supplementary Table S1). The MS profile indicated the potential for nicotinic acid incorporation in all eight AA analogs, however, only tyrosine (**4ab**) and tryptophan (**12ab**) analogs, with nicotinic acid incorporation in either of two possible positions, could be confirmed by MS/MS (Figure 6 and Supplementary Figure S7). With individual analog peaks strongly overlapping in the MS profile, MS/MS of two coeluting analogs as well as combined peak area were used for further structure assignment and relative quantification, respectively.

The relative amounts of the most common AA and nicotinic acid analogs, asperphenidines Y1–Y2 (**4ab**), and asperphenidines W1–W2 (**12ab**), were further compared to those of the asperphenidines F1–F2 (**1ab**) (Figure 7B). The relative production of asperphenidines followed the same pattern as observed in asperphenamate production: asperphenidines F1–F2 being the major nicotinic acid analogs, followed by asperhenidines Y1–Y2 and W1–W2. Moreover, production of all three upon growth on tryptophan supplemented media was drastically higher in comparison to control, with four- and five-fold increase in aspherphenidines F1–F2 and Y1–Y2, and 12-fold increase for asperphenidine W1–W2 production. However, upon anthranilic acid supplement, a significant increase was observed only in asperphenidine F1–F2 production. Additionally, production of all asperhenamates and asperhenidines was slightly lower to that of the control. Additional analysis of other AA supplement cases or additional inorganic nitrogen supplement experiments did not trigger similar nicotinic acid incorporation response.

### *Para*-Substituted Phenylalanines Are Incorporated in Either of N-Benzoyl Amino Acid Moieties

**FIGURE 8:**
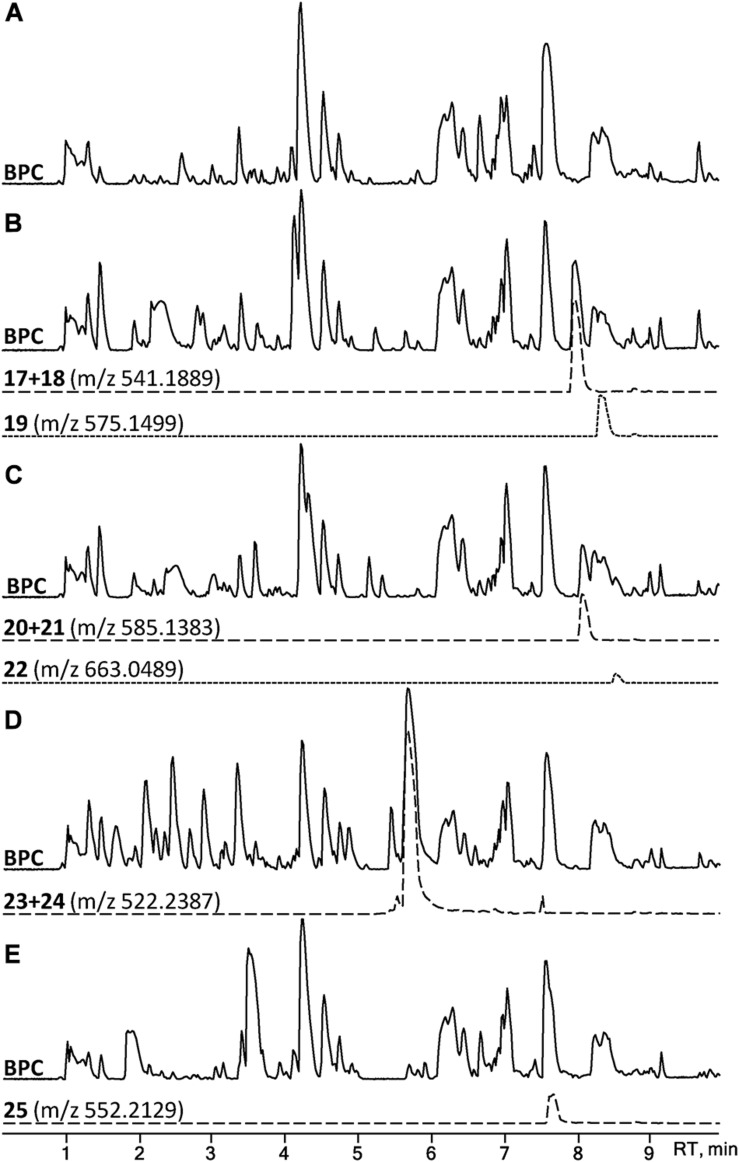
HPLC-MS analysis for asperphenamate analog production upon para-substituted phenylalanine supplement on CZ media. BPCs of *P. astrolabium* extracts of **(A)** control and growth **(B)** chloro-, **(C)** bromo-, **(D)** amino-, and **(E)** nitro- phenylalanine supplemented media. EIC in dashed line corresponds for a single amino acid, while dotted indicates a double amino acid incorporation.

**FIGURE 9:**
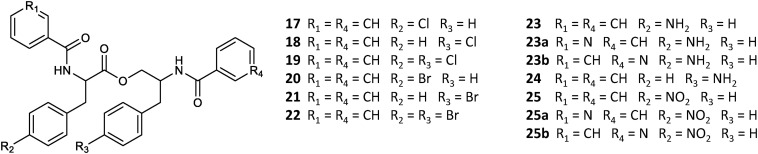
Structural overview of HRMS/MS characterized asperphenamate analogs from *P. astrolabium* upon para-substituted phenylalanine supplement study.

A set of four *para*-substituted, namely chloro-, bromo-, amino- and nitro-, phenylalanines were used to investigate the uptake of non-natural phenylalanines in asperphenamate biosynthesis. The targeted MS analysis was performed by search of masses corresponding to a single or double AA exchange in both asperphenamate and asperphenidine backbones (Supplementary Table S1). This revealed, that incorporation of single *para*-substituted AA was successful in all four supplement cases, however, a double *para*-substituted AA exchange was also observed in the halogenated phenylalanine supplement experiments (Figure 8). Moreover, small amounts of asperphenidine derivatives for single amino- and nitro-prenylalanine incorporation analogs were also detected. Subsequent MS/MS analysis revealed, that halogenated *para*-substituted phenylalanine can be incorporated at either or both reduced or non-reduced parts of the molecule, resulting in three analogs each for chloro- (**17–19**) and bromo- (**20–22**) asperphenamates (Figure 9 and Supplementary Figure S8). In case of amino- and nitro- phenylalanine exchange, the single AA incorporation was clearly preferred at the non-reduced part of the molecule, with only trace amounts of substituted AA incorporation at the reduced part of the molecule detected (**23–25**). As a result, asperphenidine analogs were detected only with AA exchange at the non-reduced part of the molecule, resulting in two analogs each for amino- (**23ab**) and nitro – (**25ab**) asperphenidines (Figure 9 and Supplementary Figure S9). Additionally, non-reduced and reduced pathway intermediates, containing *para*-substituted phenylalanines, were also detected in each of the supplement study cases.

### Asperphenamate Amino Acid Exchange Is Also Observed in Other Section *Brevicompacta* Strains

To compare the production of asperphenamate analogs among section *Brevicompacta*, the chemical profile of *P. astrolabium* was compared to three other section species, namely *P. olsonii*, *P. bialowiezense*, and *P. brevicompactum* (Houbraken et al., 2020). Targeted MS search based on 5 readily observed asperphenamates in *P. astrolabium* (asperphenamates F, Y, W, L, and asperphenidine F1) revealed, that all the other strains were also able to exchange phenylalanine in the non-reduced AA moiety (Supplementary Figure S10). Additionally, all three strains were producing isoleucine analog in similar or higher relative amount in comparison to asperphenamate L and were able to produce compound **5**, which was not detected in *P. astrolabium*. Moreover, *P. brevicompactum* revealed two peaks corresponding to the value of Asperphenamate W protonated adduct (m/z 546.2385), both with the same fragmentation patterns, suggesting them being structural isomers.

### Amino Acid Exchange Affects the Asperphenamate Analog Cytotoxicity

Asperphenamates F, Y, W, and L (**1**, **4**, **12** and **13**) as well as aspephenidine F1 (**1a**) were tested for their cytotoxic activities against five cancer cell lines, i.e., lung carcinoma A549, breast adenocarcinoma MCF7, skin melanoma A2058, hepatocyte carcinoma HepG2 and pancreas carcinoma MiaPaca (Table 2). Asperphenamate Y and Asperphenidine F1 were the only compounds exhibiting moderate cytotoxic activities against MCF7 and MiaPaca cell lines, respectively. Asperphenamates F and Y exhibited moderate activity against HepG2 cell lines, whereas all but asperphenamate L showed activity toward A2058 cell line, with asperphenamate F exhibiting the strongest activity. Asperphenamate L did not show activity against any of the cell lines at the tested concentrations. None of the compounds exhibited activity against A549 cell line at tested concentrations.

**TABLE 2:** Cytotoxic activities of 1, 1a, 4, 12, and 13.

Compound	EC_50_ (μg/mL)				
	A549	MCF7	A2058	HepG2	MiaPaca
1	>46	>46	1.1	28.5	>46
4	>46	23	24.8	21.6	>46
12	>46	>46	16.6	>46	>46
13	>46	>46	>46	>46	>46
1a	>46	>46	13.3	>46	13.3

*A549, lung adenocarcinoma; MCF7, breast carcinoma; A2058, skin melanoma; HepG2, hepatocellular carcinoma; MiaPaca, pancreas carcinoma.*

## Discussion

Only a handful of asperphenamate analogs from fungal sources, including endophytic and parasitic fungi, have been isolated to date (Jia et al., 2009; Frisvad et al., 2013; Houbraken et al., 2020). In this study, we have demonstrated yet another powerful application of a HRMS/MS guided discovery approach, for detection and structural characterization of novel peptide natural products via HRMS/MS fragmentation pattern analysis, which is in line with current HRMS/MS based peptide detections and characterization approaches (Mohimani et al., 2017; Jarmusch et al., 2020; Ricart et al., 2020). Moreover, we report that the choice of complex growth medium and/or a simple growth media supplement with selected building blocks, such as proteogenic and non-proteogenic AAs, *P. astrolabium* and related species can produce a series of novel asperphenamate analogs, which can be readily characterized by HRMS/MS.

Incubation of *P. astrolabium* IBT 28865 on complex media revealed the strain being readily capable of exchanging phenylalanine for tyrosine, leucine or tryptophan in N-benzoylphenylalanine moiety, suggesting that the preferred AA substrate should be either aromatic or aliphatic. A subsequent proteogenic AA supplement study supported the hypothesis, with all but one AA incorporated being either aromatic or aliphatic. It can be speculated, that the incorporation of AA is dependent on the side chain size and conformational similarity to that of phenylalanine, since tyrosine, leucine and methionine have the highest production rate in the AA supplement studies, whereas the smaller alanine and isoleucine analogs are produced at the lower rate. Lastly, histidine was the only non-hydrophobic AA to be incorporated into the asperphenamate backbone, something that can be attributed to its similarity to the other aromatic AAs, thereby likely interacting via similar π–π interactions.

Although the relative production upon tyrosine supplement decreased in comparison to the control, it might be attributed to tyrosine being preferentially taken up by other pathways, such as di- or tetra-peptide biosynthesis. Nevertheless, upon growth on non-supplemented media, it was observed that tyrosine incorporation in general was preferred over any other AA incorporation. Subsequent supplement study with other synthetic *para*-substituted phenylalanines confirmed previous observation, with all four selected substrates being incorporated into the asperphenamate backbone irrespective of the size of the *para*-moiety. In comparison to proteogenic AAs being mainly incorporated into the non-reduced part of asperphenamate backbone, *para*-substituted AAs were readily incorporated into either or both the reduced and the non-reduced part of the molecule. Additionally, no other pathway intermediates rather than N-benzoylphenylalanine, N-benzoylphenylalaninol and respective para-substituted phenylalanine analogs were observed. This suggests, that only intermediates with the highest similarity to phenylalanine intermediates can be recognized and released from the asperphenamate biosynthetic machinery.

The production of nicotinic acid containing analogs, asperphenidines, was strongly correlated with production of the corresponding asperphenamates upon growth on complex media. However, upon proteogenic AA supplement study, asperphenidines F1–F2 and Y1–Y2 were produced in higher amounts when tryptophan was supplemented, and in case of asperphinidines F1–F2 also in anthranilic acid supplemented media. This suggest, that biosynthesis of asperphenamate directly intercepts primary metabolism, since both anthranilic and nicotinic acids are intermediates of tryptophan catabolism in nicotinamide adenine dinucleotide (NAD) biosynthesis (Foster and Moat, 1980). This hypothesis can be further substantiated by the fact that no other organic and inorganic nitrogen source resulted in similar nicotinic acid production and incorporation response. However, no incorporation of directly supplemented benzoic acid derivatives, namely 4-hydroxybenzoic and anthranilic acids, was observed. This indicated, that incorporation of nicotinic acid is most likely driven by the availability of the substrate, rather than promiscuity of either of NRPS domains, since there are only a minute structural difference among benzoic and nicotinic acids, as well as no clear discrepancies among preference of nicotinic acid over benzoic acid by either of the NRPS domains.

In general natural product biosynthesis of small peptides involves a very strict uptake of AAs controlled by the NRPS adenylation domains leading to a conserved sequence of AAs present in the final product (Fischbach and Walsh, 2006). However, certain cyanobacteria have been reported to possess adenylation domains capable of activation of two or more chemically distant AA (Kaljunen et al., 2015; Meyer et al., 2016). In contrast, our study revealed an unusually high flexibility, rather than specificity of fungal adenylation domain toward the uptake of structurally related natural AAs, as well as synthetic *para*-substituted phenylalanine analogs. Such unusual NRPS flexibility is rather uncommon, with only one recent similar case observed in filamentous fungi (Hai et al., 2020). Recently, the Tang lab demonstrated that the hybrid NRPS-NRPKS involved in biosynthesis of α-pyrones in *Aspergillus niger* is also promiscuous toward the uptake of tyrosine, leucine and a number of *para-*substituted phenylalanines with small substitution groups (Hai et al., 2020). However, with the higher variety of natural AA being tolerated in asperphenamate biosynthesis, our results altogether suggest an even more relaxed substrate specificity in comparison to that of α-pyrone biosynthesis.

Interestingly, three other related species from section *Brevicompacta, P. olsonii*, *P. bialowiezense*, and *P. brevicompactum*, were also found to be producers of the same analogs as observed in *P. astrolabium* when grown on complex media. In addition, detection of several other asperphenamates, such as a 4-hydroxybenzoic acid containing analog (**5**), indicates an even more relaxed substrate specificity in comparison to that of *P. astrolabium*. Nonetheless, it might be speculated that the aforementioned analogs are not observed in *P. astrolabium* due to a lower growth rate in comparison to the other three strains (Serra and Peterson, 2007; Perrone et al., 2015). Moreover, the presence of two Asperphenamate W stereoisomers in *P. brevicompactum* suggest the presence of a biosynthetically unrelated enzymatic activity responsible for epimerization of tryptophan. Similar enzymatic activity was previously characterized in a single-module NRPS responsible for specific stereoconversion of L-tryptophan to D-tryptophan in *A. niger* (Hai et al., 2019).

Asperphenamates F, Y, L, and W, as well as asperphenidine F1 were tested against five cancer cell lines. Although asperphenamate L did not exhibit activity against any of the cell lines, the four other compounds revealed moderate activities against breast, skin, liver or pancreas cell lines. In particular, asperphenamate Y was the only active compound against the breast cell line, suggesting that the presence of tyrosine at the non-reduced AA moiety might be essential for the observed activity. Therefore, further investigations of asperphenamates harboring a para-substituted phenylalanine could be of interest for future cytotoxicity studies. Moreover, asperphenidine F1, was the only active candidate against the pancreas cell line, suggesting the nicotinic acid analogs being more active than the benzoic acid analogs. Although our cytotoxicity results for asperphenamates F and Y, as well as asperphenidine F1 are comparable to the previously published data, none of them show improved bioactivity compared to synthetic asperphenamate derivatives (Li et al., 2012; Yuan et al., 2012, 2018, 2019, 2020; Liu et al., 2016, 2018).

In conclusion, HRMS/MS based analysis and the use of a targeted media supplement approach demonstrated an extraordinary relaxed substrate specificity in the double NRPS system responsible for asperphenamate production. The proteogenic and non-proteogenic *para*-substituted L-phenylalanine analog supplements led to biosynthesis of 22 new analogs, all of which could readily be characterized by HRMS/MS. Here we proposed a standardized naming system for asperphenamate and asperphenidine analogs denoting specific amino acid incorporation. This strategy illustrates the potential for future combinatorial biosynthesis of asperphenamate and similar small NRPS products.

## Data Availability Statement

The original contributions presented in the study are included in the article/Supplementary Material, further inquiries can be directed to the corresponding author/s.

## Author Contributions

KS and TL designed the experiments. KS and JF performed HRMS/MS library search. KS, XW, and FN performed purification and structure elucidation, with the assistance by CG. KS performed asperphenamate design study and subsequent HRMS/MS the data analysis compounds, assisted by TI. OG, FV, MR, and TM designed and performed the bioassay. KS wrote the manuscript with contribution from all authors.

## Conflict of Interest

The authors declare that the research was conducted in the absence of any commercial or financial relationships that could be construed as a potential conflict of interest.
