# Supplementary material for: Mass Spectrometry Guided Discovery and Design of Novel Asperphenamate Analogs From Penicillium astrolabium Reveals an Extraordinary NRPS Flexibility
Source: Front Microbiol. 2021 Jan 15;11:618730. doi: 10.3389/fmicb.2020.618730 (PMC7843940; doi:10.3389/fmicb.2020.618730)

Supplementary information

Mass spectrometry guided discovery and design of novel Asperphenamate analogues from *Penicilium astrolabium*

Karolina Subko[1], Xinhui Wang[1], Frederik H. Nielsen[1], Charlotte H. Gotfredsen[2], Francisca Vicente[3], Jens C. Frisvad[1], and Thomas O. Larsen[1*]

[1]Department of Biotechnology and Biomedicine, Technical University of Denmark, Kgs. Lyngby, Denmark;
[2]Department of Chemistry, Technical University of Denmark, Kgs. Lyngby, Denmark;
[3]Fundación MEDINA, Avda del Conocimiento, 34, 18100 Armilla, Granada, Spain;

*** Correspondence:** Thomas O. Larsen, tol@bio.dtu.dk

Table of contents

Supplementary Table S1: m/z values of protonated adduct of potential Asperphenamate analogues.
Supplementary Table S2: NMR data for Asperphenamate Y (**4**).
Supplementary Table S3: NMR data for Asperphenamate W (**12**).
Supplementary Table S4: NMR data for Asperphenamate L (**13**).
Supplementary Table S5: NMR data for Asperphenamate F (**3**) and Asperphenidine F1 (**3a**).
Supplementary Figure S1: NMR spectra for Asperphenamate F (**3**).
Supplementary Figure S2: NMR spectra for Asperphenamate Y (**4**).
Supplementary Figure S3: NMR spectra for Asperphenamate W (**12**).
Supplementary Figure S4: NMR spectra for Asperphenamate L (**13**).
Supplementary Figure S5: ^{1}H NMR spectrum for Asperphenidine F1 (**3a**).
Supplementary Figure S6: MS/MS spectra and assignment of natural Asperphenamate analogues.
Supplementary Figure S7: MS/MS spectra and assignment of Asperphenidines Y1-Y2, W1-W2.
Supplementary Figure S8: MS/MS spectra and assignment of chloro- and bromo-phenylalanine asperphenamate derivatives
Supplementary Figure S9: MS/MS spectra and assignment of amino- and nitro -phenylalanine asperphenamate and asperphenidine derivatives.
Supplementary Figure S10: BPCs of selected fungi from *Brevicompacta* section.

Supplementary Table 1: m/z values of protonated adduct of potential asperphenamate analogues.

		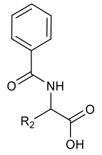<br>**(A)** R_2 = AA	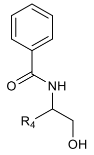<br>**(B)** R_4 = AA	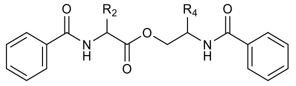<br>**(C)** R_2 = AA R_4 = Phe<br>**(D)** R_2 = Phe R_4 = AA<br>**(E)** R_2 = R_4 = AA		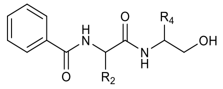<br>**(F)** R_2 = AA R_4 = Phe<br>**(G)** R_2 = Phe R_4 = AA<br>**(H)** R_2 = R_4 = AA		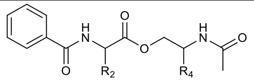<br>**(I)** R_2 = AA R_4 = Phe<br>**(J)** R_2 = Phe R_4 = AA<br>**(M)** R_2 = R_4 = AA<br>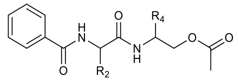<br>**(K)** R_2 = AA R_4 = Phe<br>**(L)** R_2 = Phe R_4 = AA<br>**(N)** R_2 = R_4 = AA		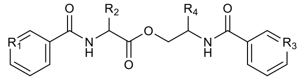<br>**(O)** R_1 = N R_2 = AA R_3 = CH R_4 = Phe<br>**(P)** R_1 = N R_2 = Phe R_3 = CH R_4 = AA<br>**(Q)** R_1 = CH R_2 = AA R_3 = N R_4 = Phe<br>**(R)** R_1 = CH R_2 = Phe R_3 = N R_4 = AA<br>**(S)** R_1 = R_3 = N R_2 = AA, R_4 = Phe<br>**(T)** R_1 = R_3 = N R_2 = Phe, R_4 = AA	
**No.**	**AA**	**(A)**	**(B)**	**(C)/(D)**	**(E)**	**(F)/(G)**	**(H)**	**(I)/(J)/(K)/(L)**	**(M)/(N)**	**(O)/(P)/(Q)/(R)**	**(S)/(T)**
**1**	Phe	270.1125	256.1332	507.2278		403.2016		445.2122		508.2231	509.2183
**2**	Tyr	285.1001	272.1281	523.2227	539.2177	419.1965	435.1914	461.2071	477.2020	524.2180	525.2132
**3**	Trp	309.1234	295.1441	546.2387	585.2496	442.2125	481.2234	484.2231	523.2340	547.2340	548.2292
**4**	Leu/Ile	236.1281	222.1489	473.2435	439.2591	369.2173	335.2329	411.2278	377.2435	474.2387	475.2340
**5**	Ala	194.0812	180.1019	431.1965	355.1652	327.1703	251.1390	369.1809	293.1496	432.1918	433.1870
**6**	Gly	180.0655	166.0863	417.1809	327.1339	313.1547	223.1077	355.1652	265.1183	–	–
**7**	His	260.1030	246.1237	497.2183	487.2088	393.1921	383.1826	435.2027	425.1932	498.2136	499.2088
**8**	Val	222.1125	208.1332	459.2278	411.2278	355.2016	307.2016	397.2122	349.2122	460.2231	461.2183
**9**	Met	254.0845	240.1053	491.1999	475.1720	387.1737	371.1458	429.1843	413.1563	492.1952	493.1904
**11**	Cys	226.0532	212.0740	463.1686	419.1094	359.1424	315.0832	401.1530	357.0937	–	–
**12**	Ser	210.0761	196.0968	447.1914	387.1551	343.1652	283.1288	385.1758	325.1394	–	–
**13**	Thr	224.0917	210.1125	461.2071	415.1864	357.1809	311.1601	399.0914	353.1707	–	–
**14**	Asn	237.0870	223.1077	474.2023	411.1769	370.1761	337.1506	412.1867	379.1612	–	–
**15**	Gln	251.1026	237.1234	488.2180	469.2082	384.1918	365.1819	426.2023	407.1925	–	–
**16**	Lys	251.1390	237.1598	488.2544	469.2809	384.2282	365.2547	426.2387	407.2653	–	–
**17**	Arg	279.1452	265.1659	516.2605	525.2932	412.2343	421.2670	454.2449	463.2776	–	–
**18**	Pro	220.0968	206.0968	457.2122	407.1965	353.1860	277.1547	395.1965	345.1809	–	–
**19**	Glu	252.0866	238.1074	489.2020	471.1762	385.1758	337.1500	427.1864	409.1605	–	–
**20**	Asp	238.0710	224.0917	475.1864	443.1449	371.1601	339.1187	413.1707	381.1292	–	–
**21**	p-Cl-Phe	304.0735	290.0942	541.1889	575.1499	437.1626	471.1237	479.1732	513.1542	542.1841	543.1794
**22**	p-Br-Phe	348.0230	334.0437	585.1383	663.0489	481.1121	559.0226	523.1227	601.0332	586.1336	587.1288
**23**	p-NH_2-Phe	285.1234	271.1441	522.2387	537.2496	418.2125	433.2234	460.2231	494.1841	523.2340	524.2292
**24**	p-NO_2-Phe	315.0978	301.1183	552.2129	597.1980	448.1867	463.1976	490.1973	535.1823	553.2082	554.2034

Supplementary Table S2: NMR data for Asperphenamate Y (**4**) in chloroform ($CDCl_3$).

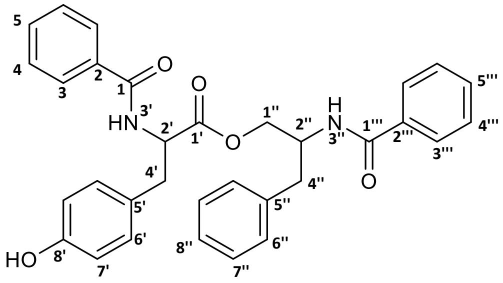

**No.**	**δ_H, mult. (*J* in Hz)**	**δ_C**	**COSY**	**H2BC**	**HMBC**
**1**		167.7			
**2**		133.4			
**3**	7.65, m	127.2	4	C-4	C-1, C-3, C-5,
**4**	7.39, m	128.8	3, 5	C-3, C-5	C-2, C-4
**5**	7.5, tt (7.4, 1.2)	132.2	4	C-4	C-3
**1'**		172.2			
**2'**	4.87, d (6.7)	54.8	3', 4'	C-4'	C-1, C-1', C-4', C-5'
**3'**	6.59, d (6.6)		2'	C-2'	C-1, C-1', C-4'
**4'**	3.20, dd (14.0, 6.5)<br>3.14, dd (14.0, 6.9)	36.9	2'	C-2'	C-1', C-2', C-5', C-6'
**5'**		127.6			
**6'**	7.05, m	130.5	7'	C-7'	C-4', C-6', C-7', C-8'
**7'**	6.76, m	116.0	6'	C-6'	C-5', C-7', C-8'
**8'**		155.3			
**1''**	4.50, dd (11.4, 3.6)<br>4.04, dd (11.4, 4.5)	65.5	2''	C-2''	C-1', C-4'', C-2''
**2''**	4.60, m	50.6	1'', 3'', 4''	C-1'', C-4''	
**3''**	6.71, d (8.4)		2''	C-2''	C-1'''
**4''**	3.01, dd(13.9, 6.5)<br>2.91, dd (13.9, 8.2)	37.4	2''	C-2''	C-1'' , C-2'', C-5'', C-6''
**5''**		137.1			
**6''**	7.22, m	129.5	7''	C-7''	C-4'',C- 8''
**7''**	7.31, m	128.9	6'', 8''	C-6'' , C-8''	C-5'', C-7'', C-6''
**8''**	7.24, m	127.0	7''	C-7''	C-6''
**1'''**		167.8			
**2'''**		134.1			
**3'''**	7.68, m	127.3	4'''	C-4'''	C-1''', C-3''', C-5'''
**4'''**	7.31, m	128.6	3''', 5'''	C-3''', C-5'''	C-2''', C-4'''

Weak correlations are underlined.

Supplementary Table S3: NMR data for Asperphenamate W (**12**) in chloroform ($CDCl_3$).

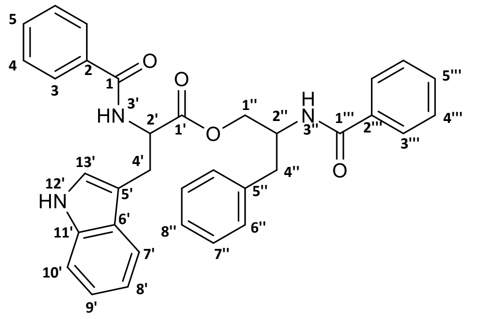

**No.**	**δ_H, mult. (*J* in Hz)**	**δ_C**	**COSY**	**H2BC**	**HMBC**
**1**		167.6			
**2**		133.5			
**3**	7.63, m	127.3	4	C-4	C-1, C-3, C-5
**4**	7.29, m	128.6	3, 5	C-3, C-5	C-2, C-3, *C*-4
**5**	7.48, t (7.4)	132.1	4	C-4	C-3, C-4
**1'**		172.5			
**2'**	5.04, q (6.5)	54.3	3', 4'	C-4'	C-1, C-1', C-4', C-5'
**3'**	6.69, d (6.4)		2'		C-1 , C-1', C-2', C-4'
**4'**	3.43, d (6.0)	27.7	2'	C-2'	C-1', C-2', C-5', C- 6', C-13'
**5'**		110.1			
**6'**		127.5			
**7'**	7.64, m	118.7	8'	C-8'	C-5', C-9', C-11'
**8'**	7.12, t (7.4)	120.1	7', 9'	C-7', C-9'	C-6', C-10'
**9'**	7.20, m	122.7	8', 10'	C-8', C-10'	C-7', C-11'
**10'**	7.33, d (7.9)	111.6	9'	C-9'	C-6', C-8'
**11'**		136.4			
**12'**	8.06, s		13'		C-5', C-6', C-13'
**13'**	7.06, d (2.2)	123.1	12'		C-4, C-5', C-6', C-11'
**1''**	4.46, dd (11.6, 3.6)<br>4.06, dd (11.6, 4.6)	65.4	1'', 2''	C-2''*	C-1', C-2'', C-4''
**2''**	4.55, m	50.6	1'' , 3'' , 4''	C-1'', C-4''	C-1''
**3''**	6.59 d (8.4)		2''		C-1'', C-2'', C-1'''
**4''**	2.94, dd (13.6, 6.7)<br>2.81, dd (13.6, 8.4)	37.3	2'', 4''	C-2''	C-1'', C-2'', C-5'', C-6''
**5''**		137.4			
**6''**	7.18, s (7.6)	129.5	7''	C-7''	C-4'', C-6'', C-7'', C-8''
**7''**	7.36, t (7.8)	128.7	6'', 8''	C-6'', C-8''	5'', 6'', 7''
**8''**	7.23, m	126.9	7''	C-7''	C-6'', C-7''
**1'''**		167.4			
**2'''**		134.4			
**3'''**	7.63, m	127.2	4''	C-4''	C-1''', C-3''', C-5'''
**4'''**	7.29, m	128.8	3''', 5'''	C-3''', C-5'''	C-2''', C-4''
**5'''**	7.43, t (7.4)	131.5	4''	C-4''	C-3''', C-4''

Weak correlations are underlined.

Supplementary Table S4: NMR data for Asperphenamate L (**13**) in chloroform ($CDCl_3$).

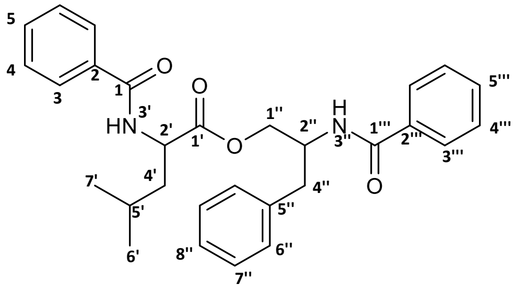

**No.**	**δ_H, mult. (*J* in Hz)**	**δ_C**	**COSY**	**HMBC**
**1**		167.8		
**2**		133.3		
**3**	7.72, m	127.1	C-4	C-1, C-3, C-5
**4**	7.41, m	128.6	C-3, C-5	C-2, C-4
**5**	7.52, m	132.2	C-4	C-3, C-4
**1'**		173.2		
**2'**	4.71, m	52.1	C-3', C-4'	C-1', C-4'
**3'**	6.46, d (6.8)		C-2'	C-1
**4'**	1.79, m<br>1.69, m	40.8	C-2', C-4', C-5'	C-1', C-2', C-5', C-6', C-7'
**5'**	1.75, m	25.1	C-4', C-6', C-7'	C-4', C-6'
**6'**	0.99, d (6.5)	22.2	C-5'	C-4', C-5', C-7'
**7'**	1.02, d(6.5)	22.8	C-5'	C-4', C-5', C-6'
**1''**	4.59, dd (11.5, 3.3)<br>4.08, dd (11.5, 4.6)	65.1	C-1'', C-2''	C-1'
**2''**	4.69, m	50.5	C-1'', C-3'', C-4''	
**3''**	6.73, d (8.2)		C-2''	C-1'''
**4''**	3.10, dd (13.6, 6.5)<br>3.01, dd (13.8, 8.2)	37.3	C-2'' , C-4''	C-1'', C-2'', C-5'', C-6''
**5''**		137.3		
**6''**	7.29, m	129.3	C-7''	C-4'', C-6'', C-8''
**7''**	7.32, m	128.7	C-6'', C-8''	C-5'', C-7''
**8''**	7.25, m	126.8	C-7''	
**1'''**		167.3		
**2'''**		134.3		
**3'''**	7.70, m	127.1	C-4'''	C-1''', C-3''', C-5'''
**4'''**	7.28, m	128.4	C-3''', C-5'''	C-2''', C-4'''
**5'''**	7.42, m	131.3	C-4'''	C-3'''

Weak correlations are underlined.

Supplementary Table S5: NMR data for Asperphenamate F (**3**) and Asperphenidine F1 (**3a**) in chloroform ($CDCl_3$).

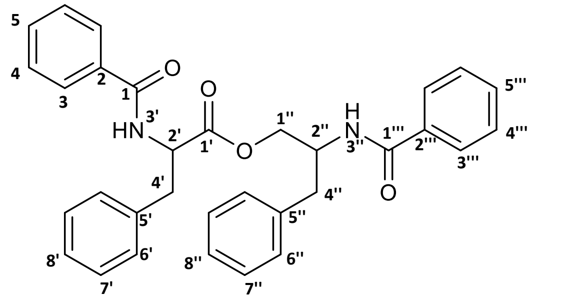

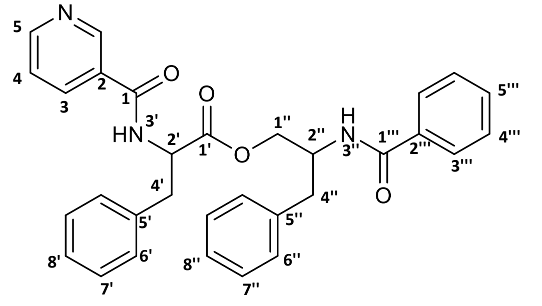

**No.**	**δ_H, mult. (*J* in Hz)**	**δ_C**	**No.**	**δ_H, mult. (*J* in Hz)**
**1**			**1**	
**2**			**2**	
**3**	7.7, dd (8.3, 1.1)	126.9	**3**	7.94, m
**4**	7.39, m	128.6	**4**	7.18-7.35, m
**5**	7.50, tt (7.5, 1.1)	131.9	**5**	8.72, dd (4.8, 1.5)
**7**				8.87, d (2.0)
**1'**			**1'**	
**2'**	4.92, q (6.6)	54.4	**2'**	4.96, q (6.7)
**3'**	6.58, d (6.6)		**3'**	6.48, d
**4'**	3.29, dd (14.0, 6.6)<br>3.21, dd (14.0, 7.0)	37.5	**4'**	3.30, dd (14.1, 6.5)<br>3.21, dd (14.0, 7.0)
**5'**			**5'**	
**6'**	7.21, m	129.1	**6'**	7.18-7.35, m
**7'**	7.29, m	128.8	**7'**	7.18-7.35, m
**8'**	7.24, m	126.7	**8'**	7.18-7.35, m
**1''**	4.54, dd (11.4, 3.4)<br>4.04, dd (11.4, 4.4)	65.3	**1''**	4.51, dd (11.4, 3.5)<br>4.09, dd (11.4, 4.1)
**2''**	4.62, m	50.2	**2''**	4.63, m
**3''**	6.67, d (8.4)		**3''**	6.59, d
**4''**	3.00, dd (13.7, 6.4)<br>2.89, dd (13.8, 8.5)	37.2	**4''**	3.00, dd (13.8, 6.5)<br>2.90, dd (13.8, 8.1)
**5''**			**5''**	
**6''**	7.23, m	129.2	**6''**	7.18-7.35, m
**7''**	7.32, m	128.3	**7''**	7.18-7.35, m
**8''**	7.25, m	127.3	**8''**	7.18-7.35, m
**1'''**			**1'''**	
**2'''**			**2'''**	
**3'''**	7.65, dd (8.3, 1.1)	127.0	**3'''**	7.66, dd (8.3, 1.0)
**4'''**	7.31, m	128.6	**4'''**	7.18-7.35, m
**5'''**	7.43, tt (7.5, 1.1)	131.3	**5'''**	7.45, m

Supplementary Figure S1: NMR spectra for Asperphenamate F (**3**).

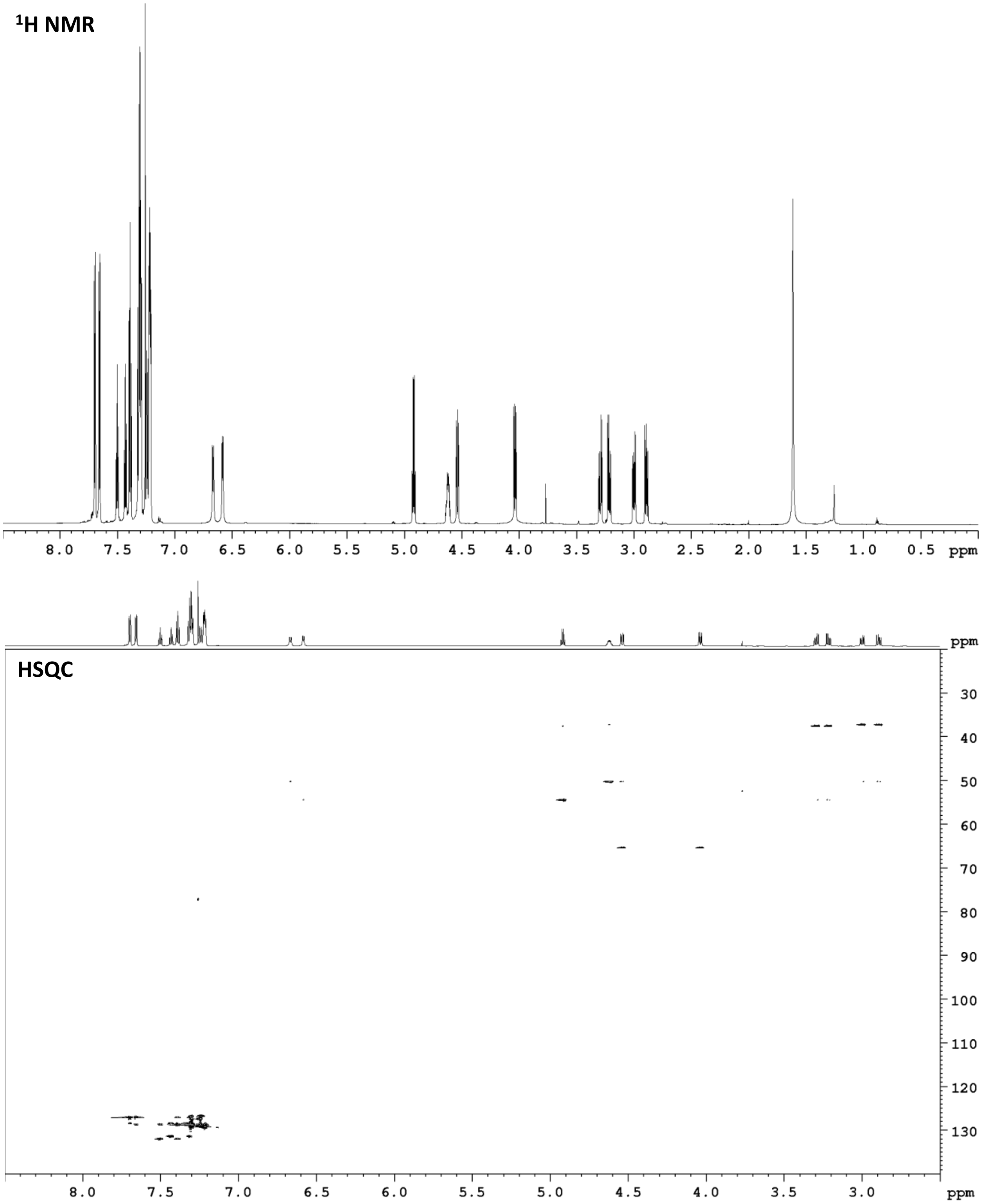

Supplementary Figure S2: NMR spectra for Asperphenamate Y (**4**).

^{1}H NMR

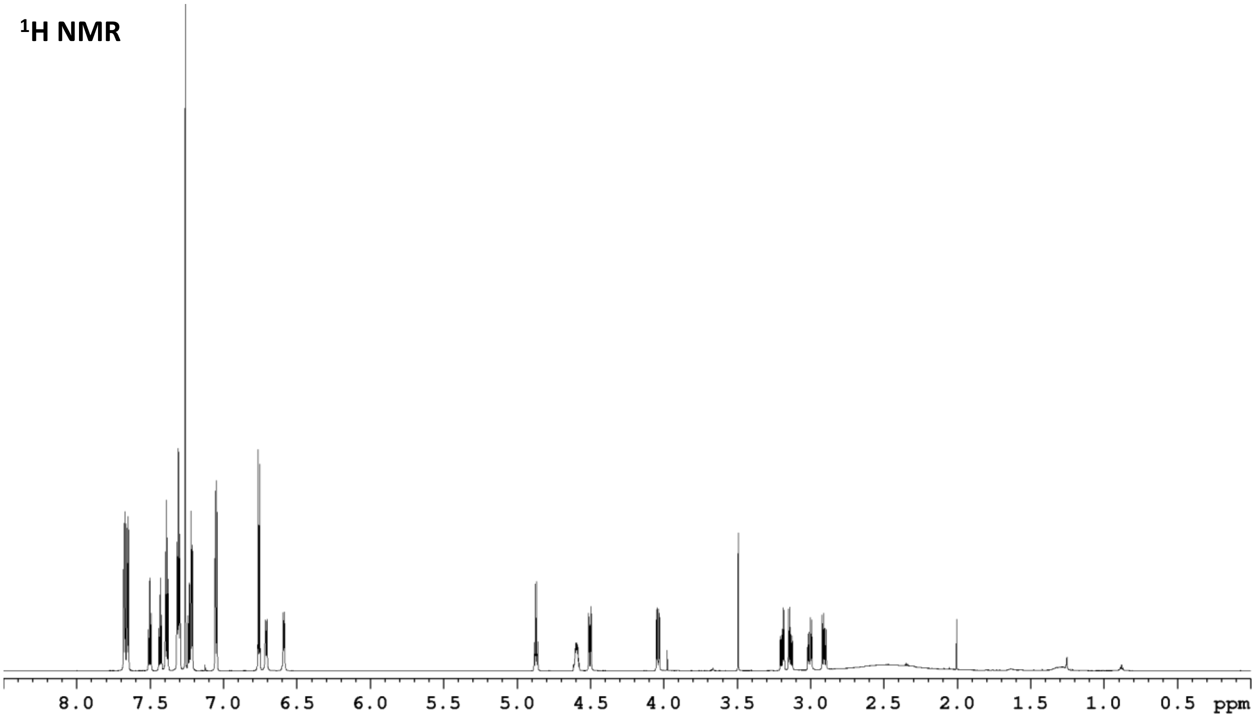

^{13}C NMR

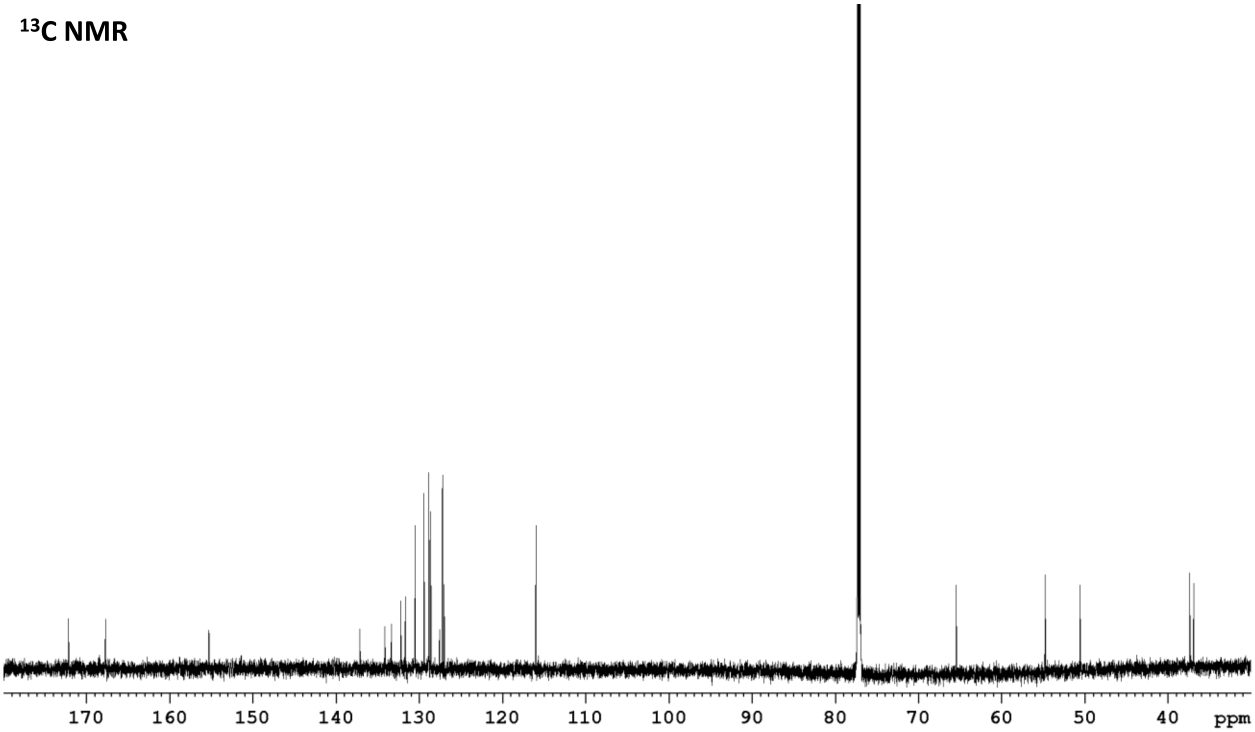

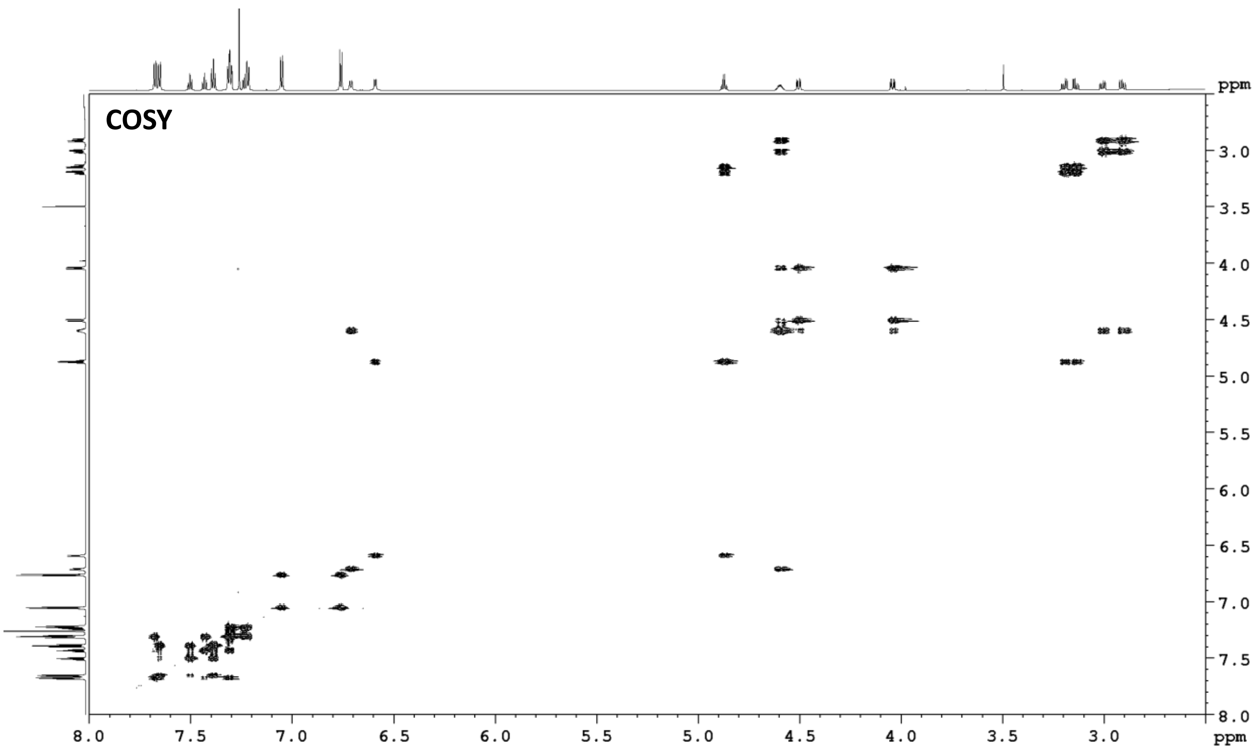
COSY
ppm
3.0
3.5
4.0
4.5
5.0
5.5
6.0
6.5
7.0
7.5
8.0
8.0 7.5 7.0 6.5 6.0 5.5 5.0 4.5 4.0 3.5 3.0

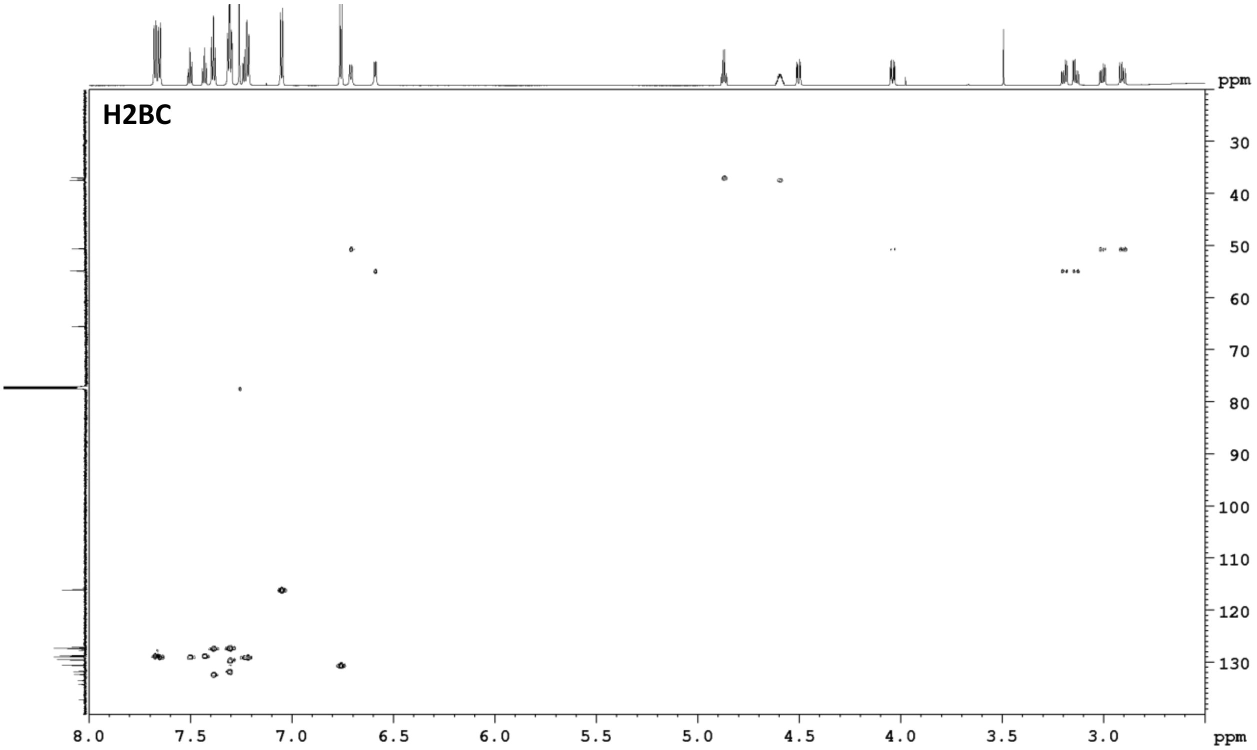
H2BC
ppm
30
40
50
60
70
80
90
100
110
120
130
8.0 7.5 7.0 6.5 6.0 5.5 5.0 4.5 4.0 3.5 3.0

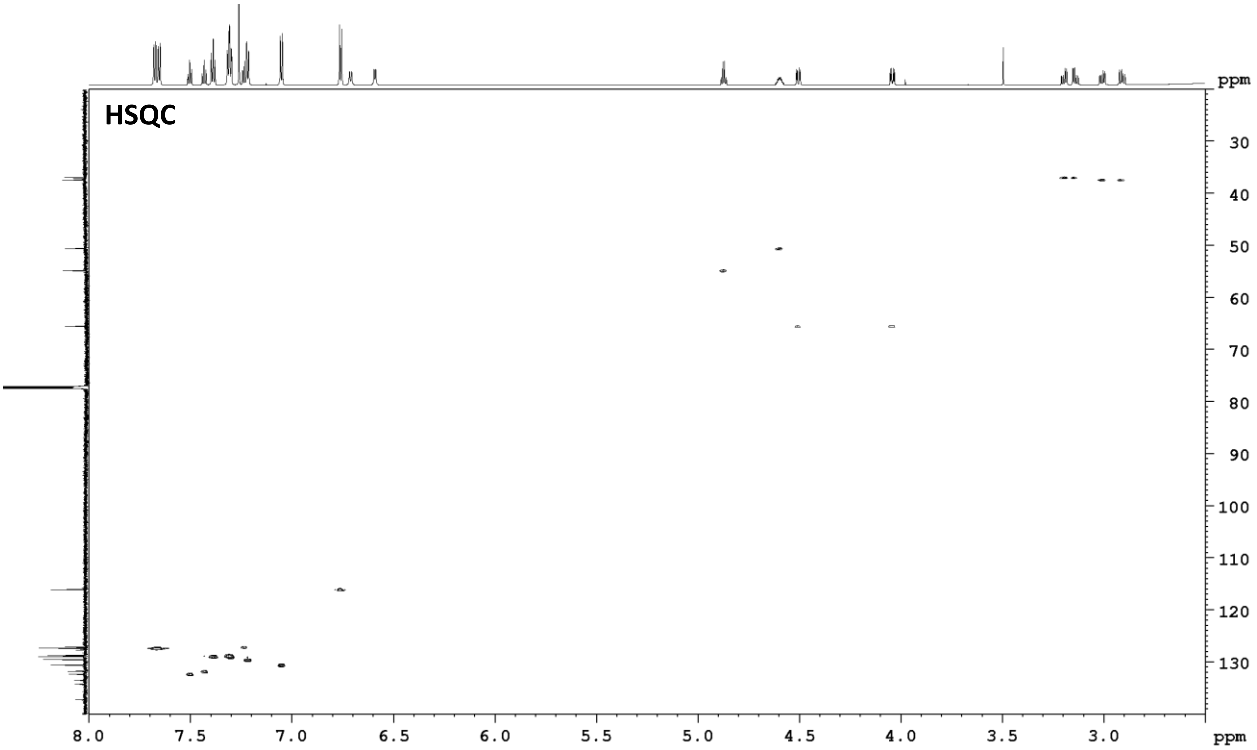
HSQC
ppm
30
40
50
60
70
80
90
100
110
120
130
8.0
7.5
7.0
6.5
6.0
5.5
5.0
4.5
4.0
3.5
3.0
ppm

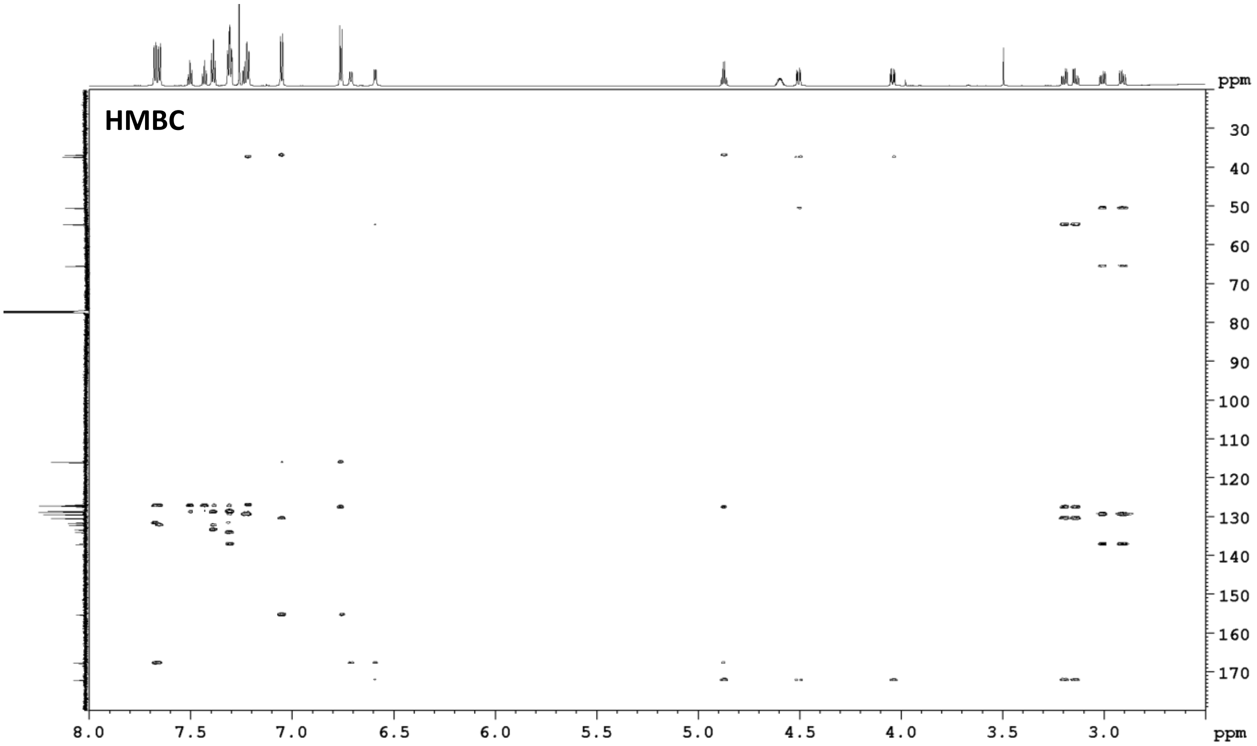
HMBC
ppm
30
40
50
60
70
80
90
100
110
120
130
140
150
160
170
8.0
7.5
7.0
6.5
6.0
5.5
5.0
4.5
4.0
3.5
3.0
ppm

Supplementary Figure S3: NMR spectra for Asperphenamate W (**12**).

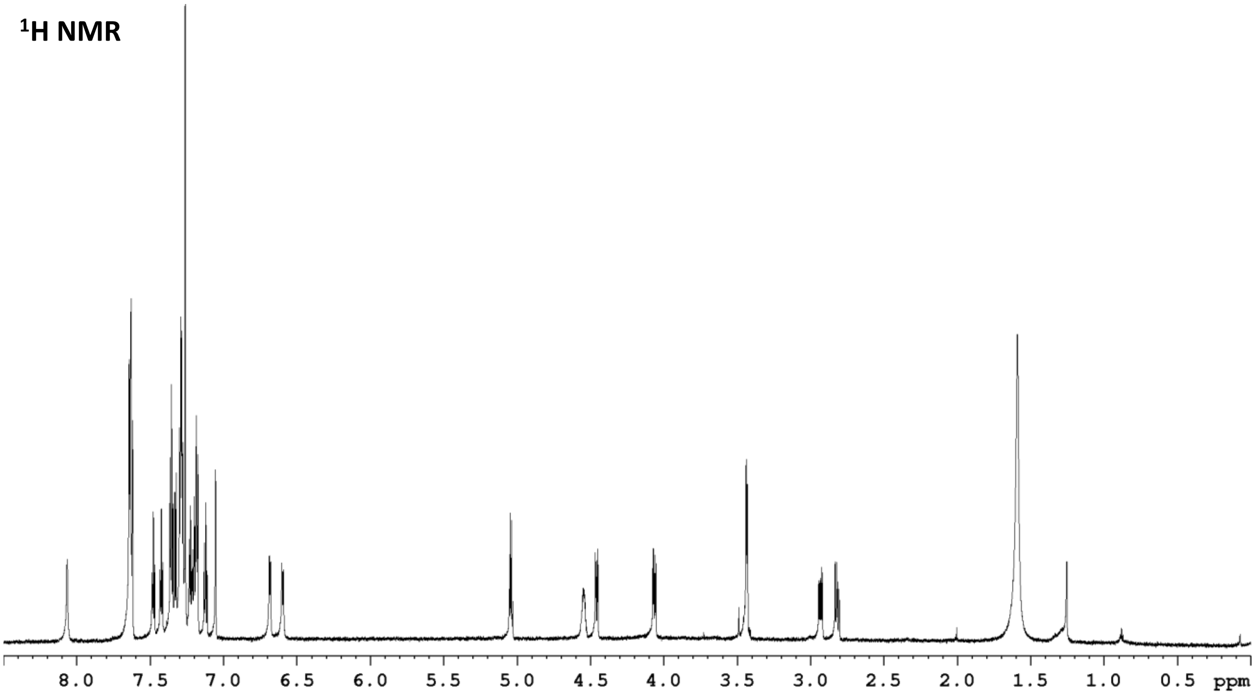

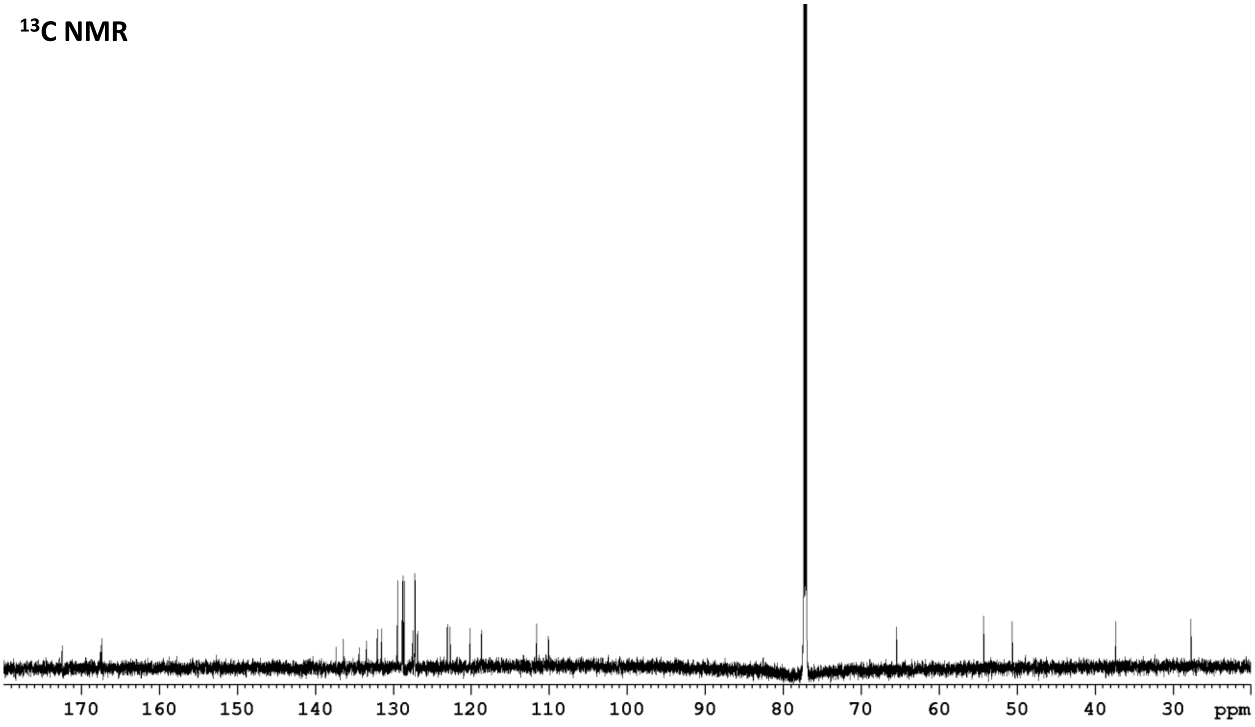

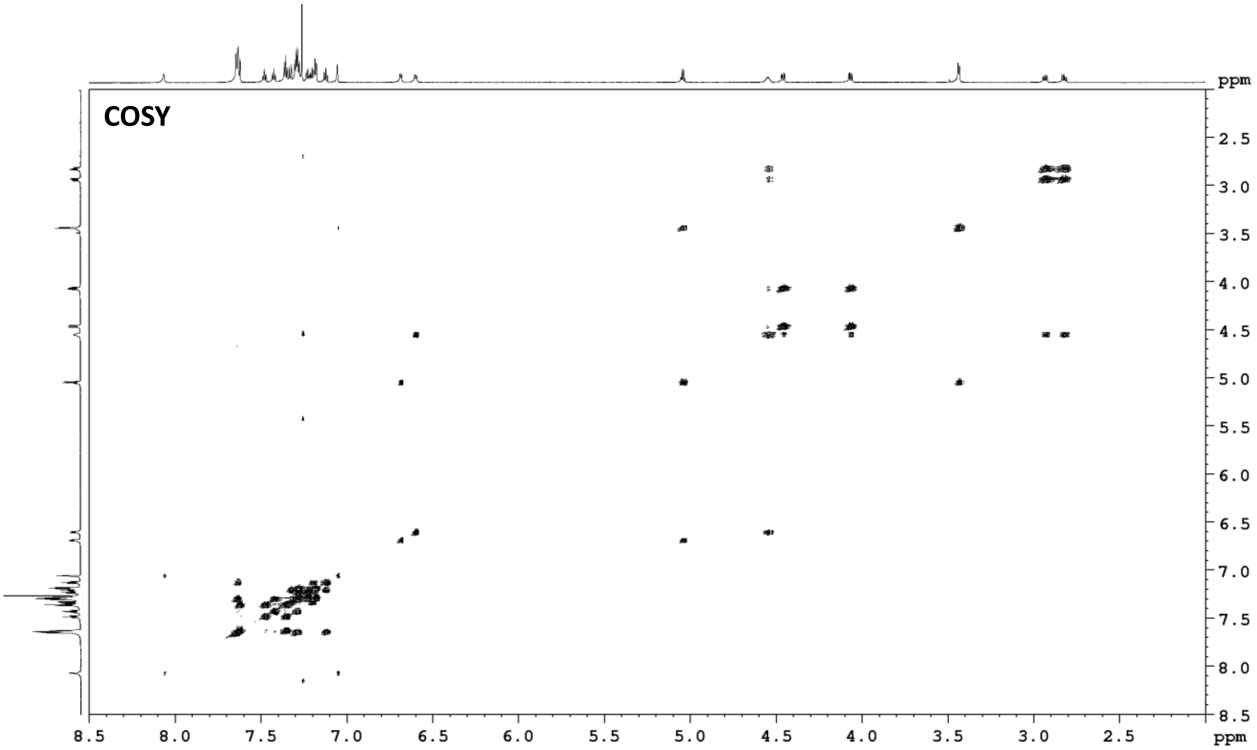
COSY
ppm
2.5
3.0
3.5
4.0
4.5
5.0
5.5
6.0
6.5
7.0
7.5
8.0
8.5
8.5 8.0 7.5 7.0 6.5 6.0 5.5 5.0 4.5 4.0 3.5 3.0 2.5
ppm

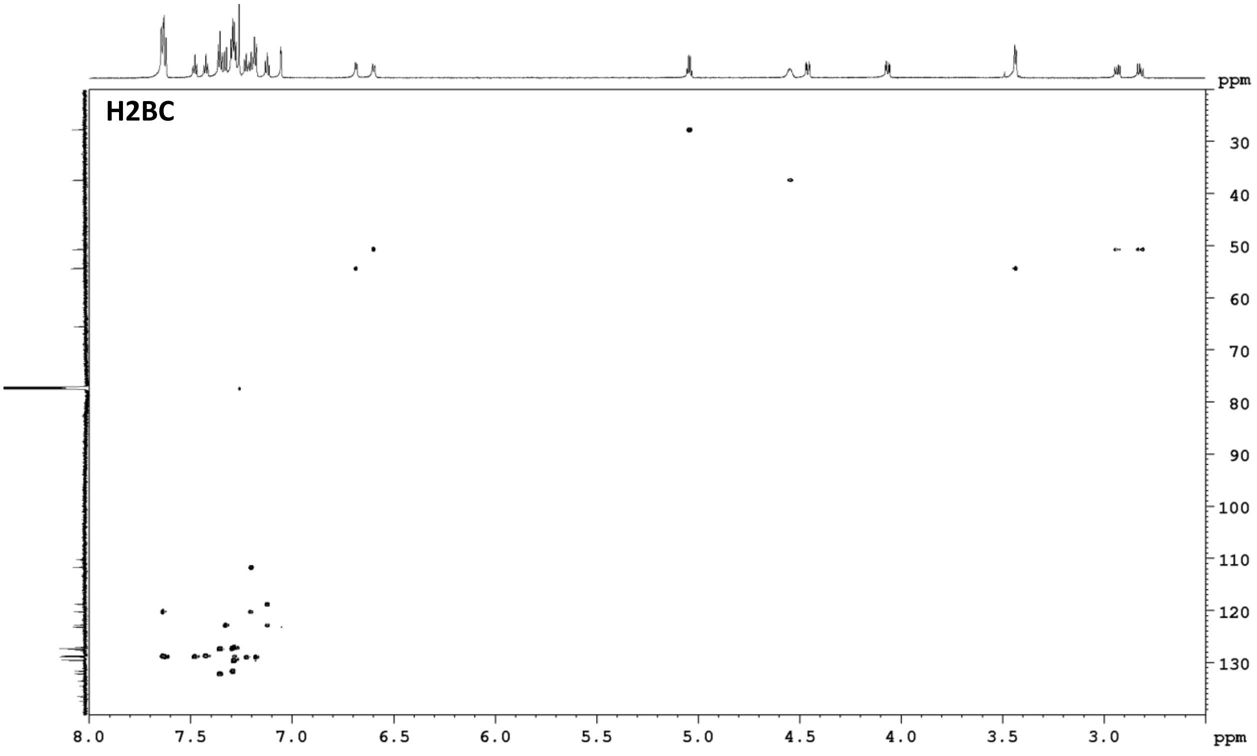
H2BC
ppm
30
40
50
60
70
80
90
100
110
120
130
8.0 7.5 7.0 6.5 6.0 5.5 5.0 4.5 4.0 3.5 3.0
ppm

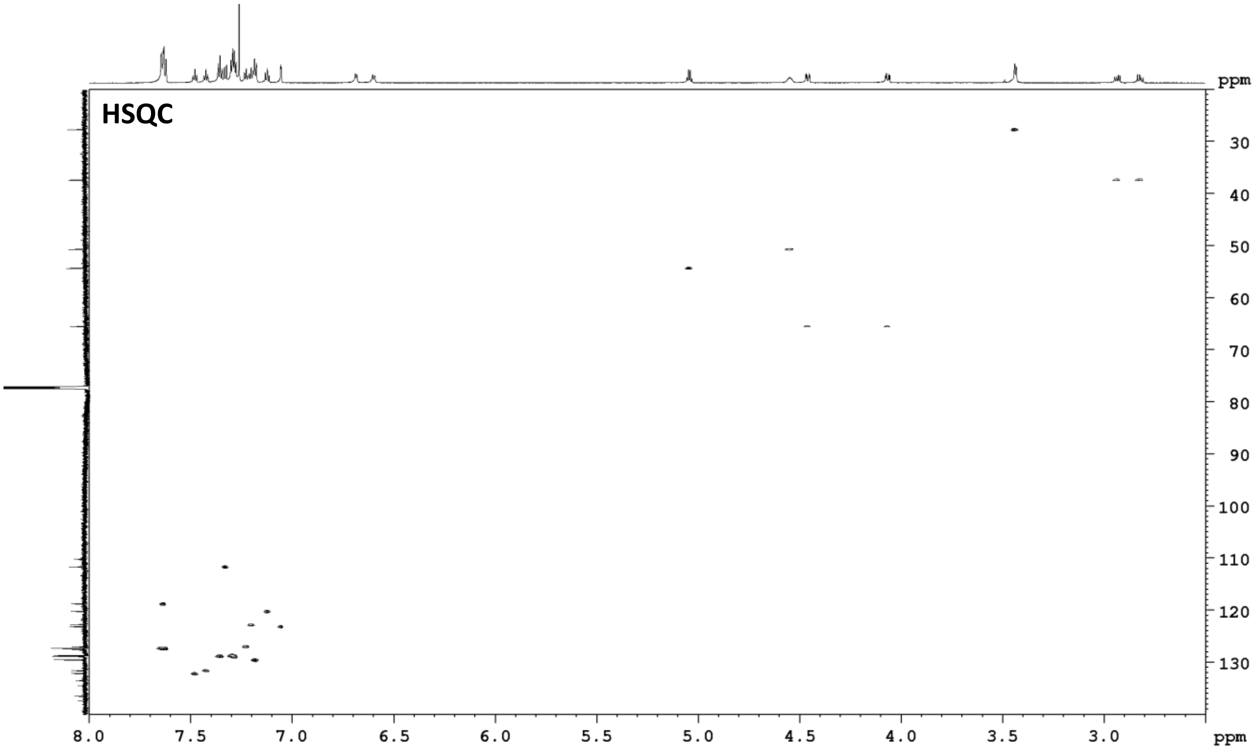
HSQC
ppm
30
40
50
60
70
80
90
100
110
120
130
8.0
7.5
7.0
6.5
6.0
5.5
5.0
4.5
4.0
3.5
3.0
ppm

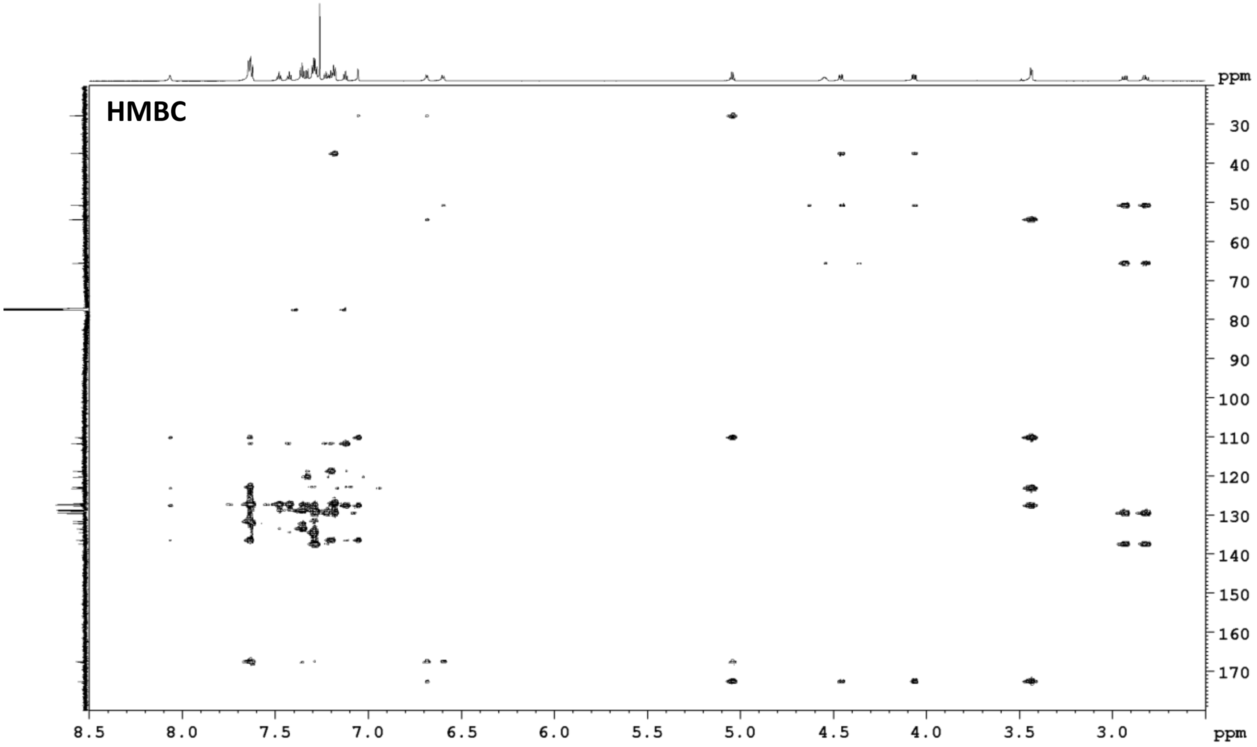
HMBC
ppm
30
40
50
60
70
80
90
100
110
120
130
140
150
160
170
8.5
8.0
7.5
7.0
6.5
6.0
5.5
5.0
4.5
4.0
3.5
3.0
ppm

Supplementary Figure S4: NMR spectra for Asperphenamate L (**13**).

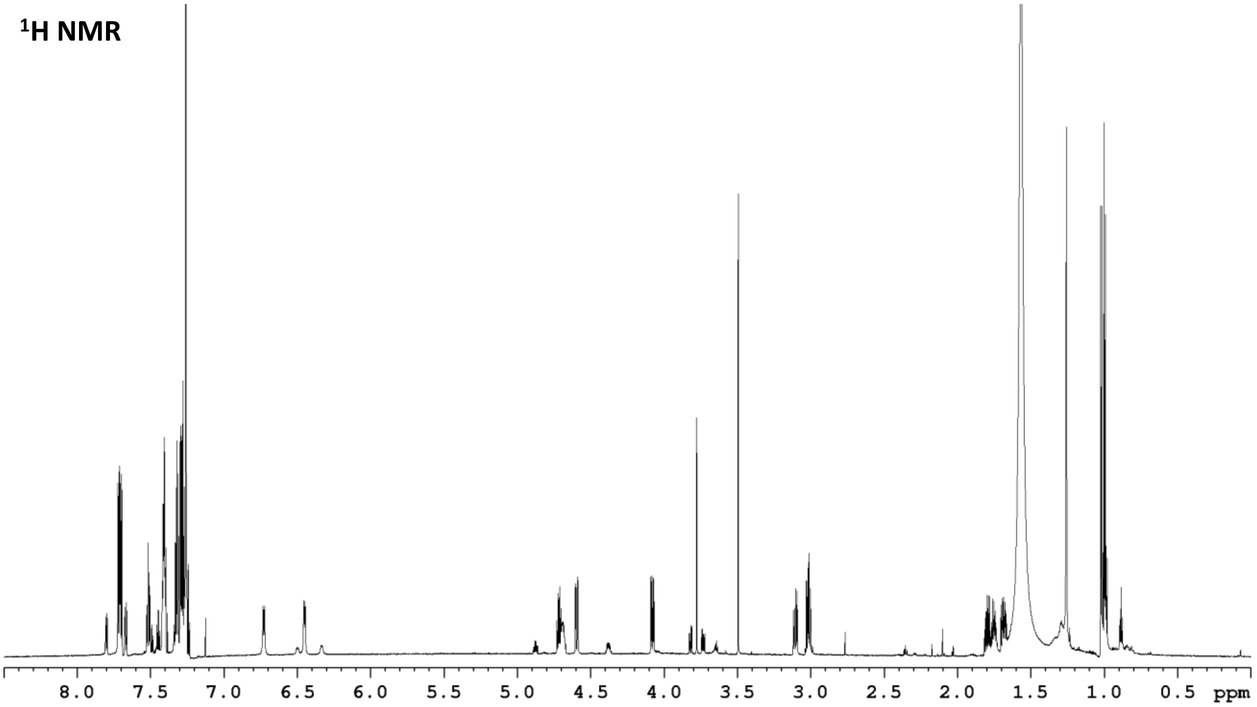

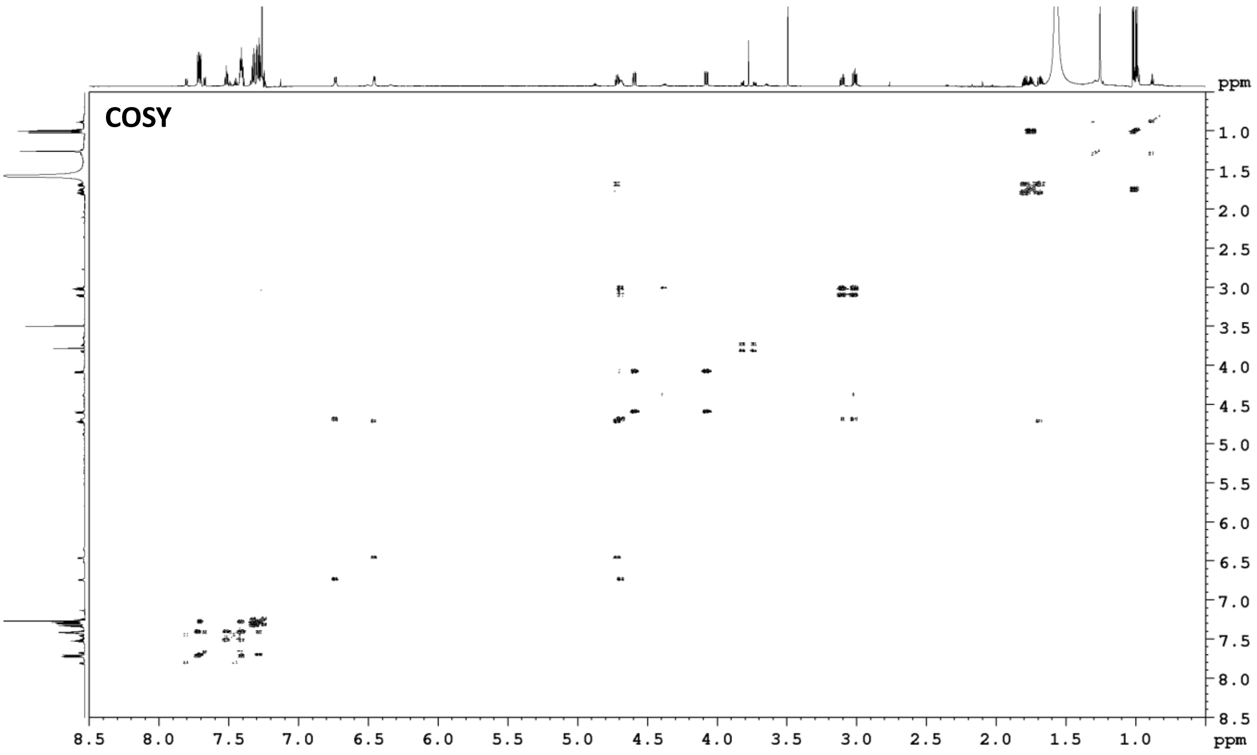

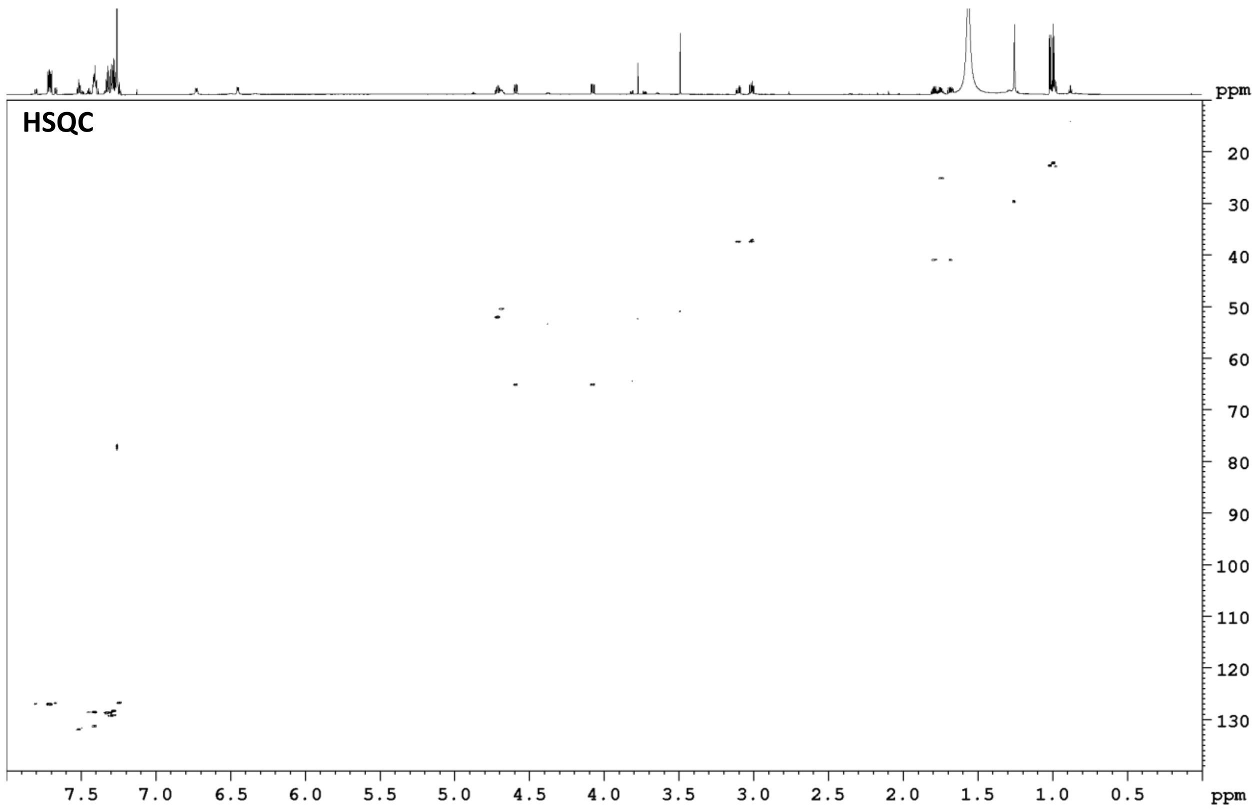
HSQC
ppm
20
30
40
50
60
70
80
90
100
110
120
130
7.5 7.0 6.5 6.0 5.5 5.0 4.5 4.0 3.5 3.0 2.5 2.0 1.5 1.0 0.5
ppm

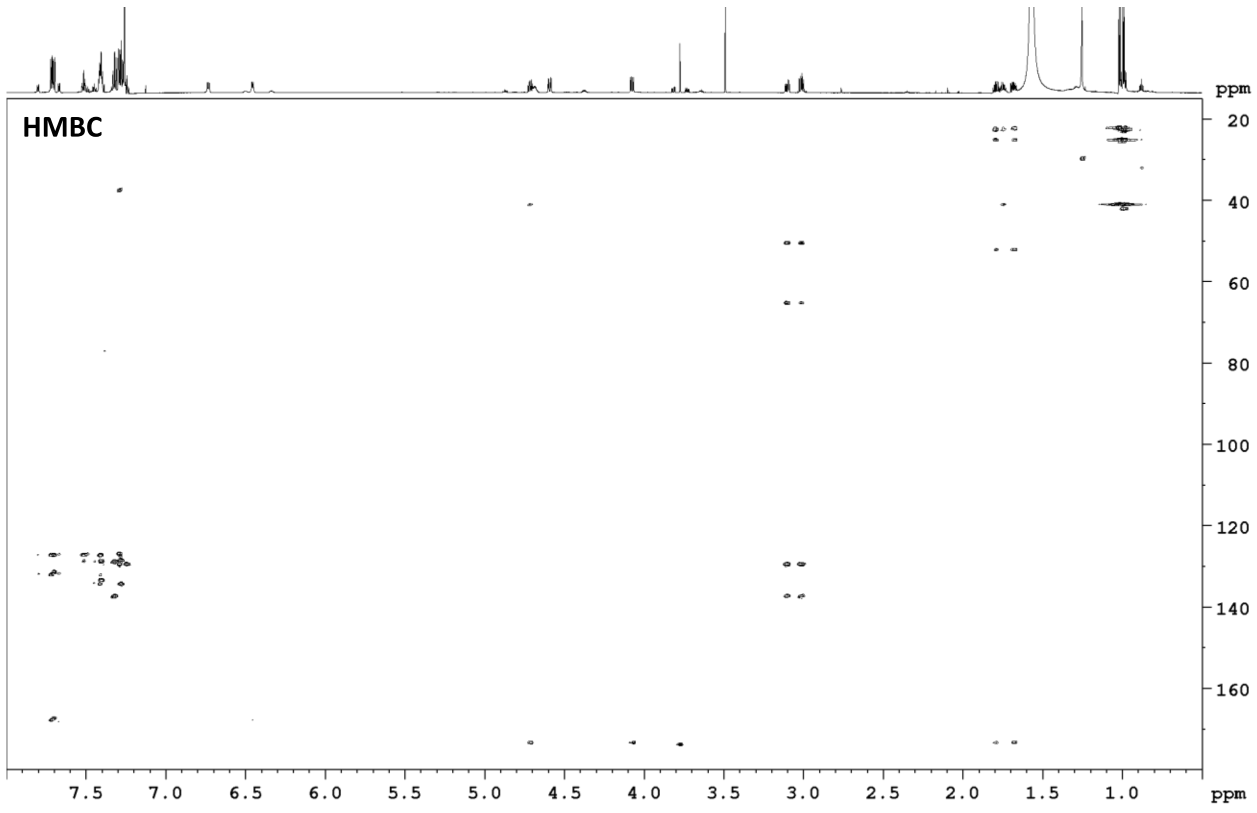
HMBC
ppm
20
40
60
80
100
120
140
160
7.5 7.0 6.5 6.0 5.5 5.0 4.5 4.0 3.5 3.0 2.5 2.0 1.5 1.0
ppm

Supplementary Figure S5: ^{1}H NMR spectrum for Asperphenidine F1 (**3a**).

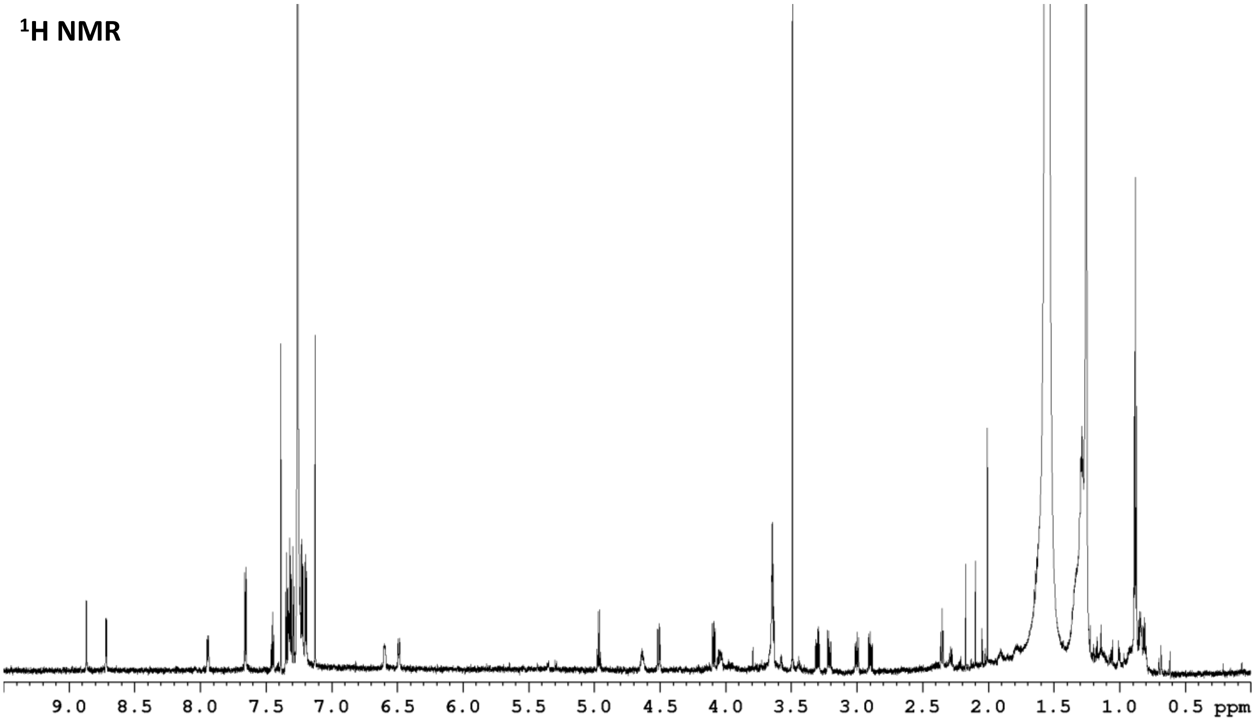

Supplementary Figure S6: MS/MS spectra and assignment of natural Asperphenamate analogues. (A) valine, (B) methionine, (C) histidine, and (D) double leucine exchange analogues.

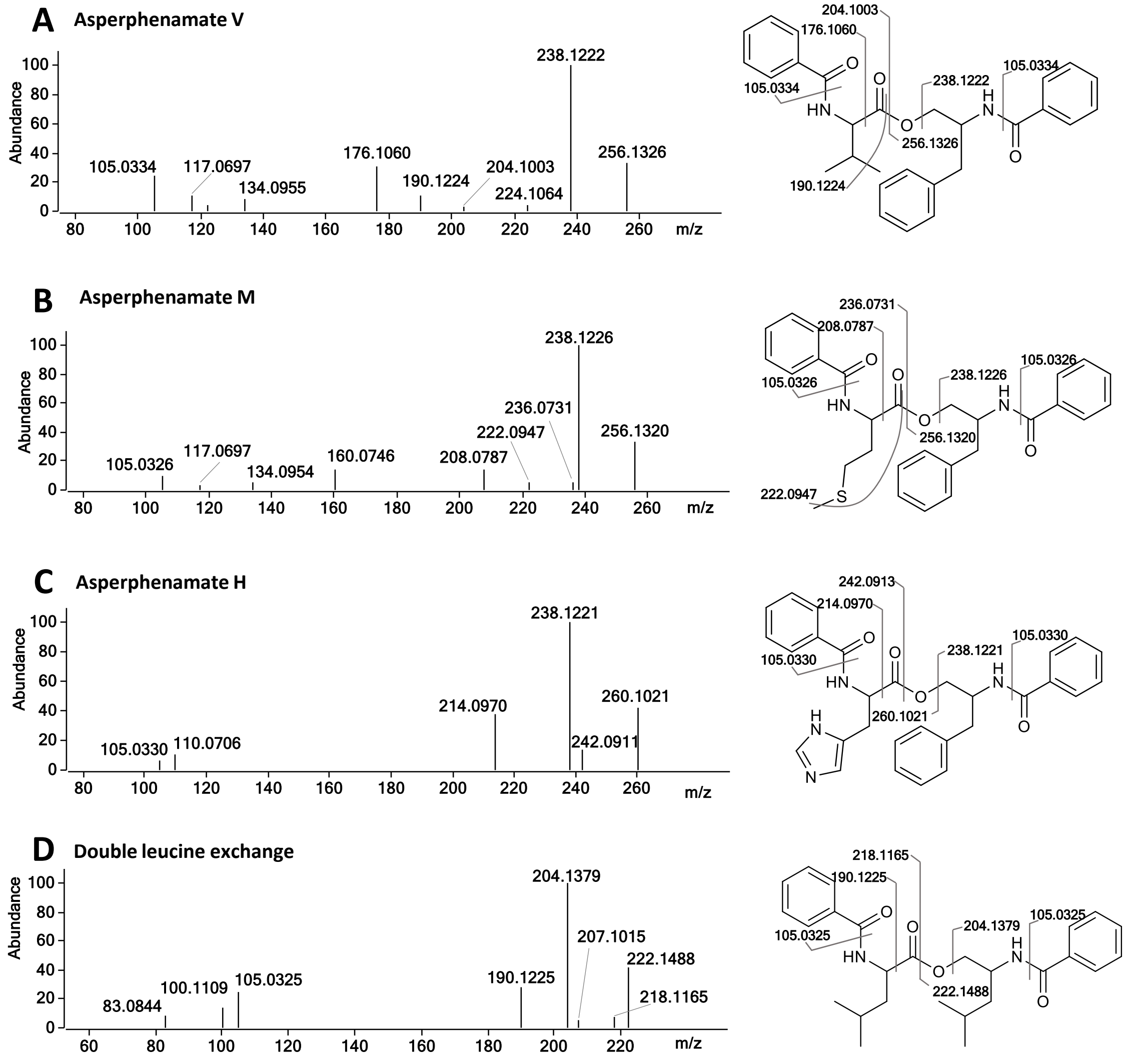

Supplementary Figure S7: MS/MS spectra and assignment of Asperphenidines Y1-Y2 (A) and W1-W2 (B). Fragment values highlighted in green correspond to nicotinic acid incorporation in the non-reduced amino acid moiety, whereas fragment values highlighted in blue correspond to nicotinic acid incorporation in the reduced amino acid moiety.

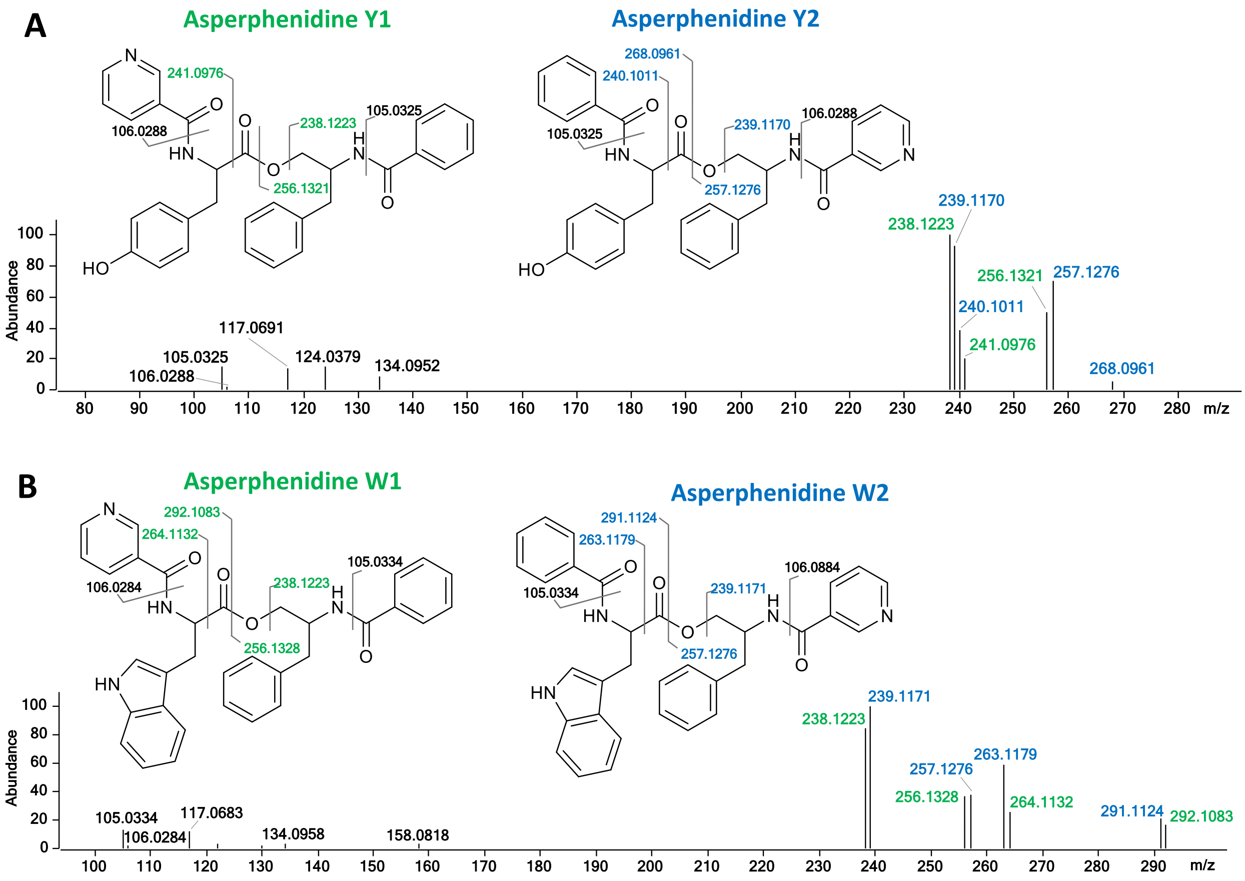

Supplementary Figure S8: MS/MS spectra and assignment of chloro- and bromo- phenylalanine asperphenamate derivatives, with single (A) and double (B) p-chloro-phenylalanine, and single (C) and double (D) p-bromo-phenylalanine incorporation. Fragment values highlighted in green correspond to substituted amino acid incorporation in the non-reduced amino acid moiety, whereas fragment values highlighted in blue correspond to substituted amino acid incorporation incorporation in the reduced amino acid moiety.

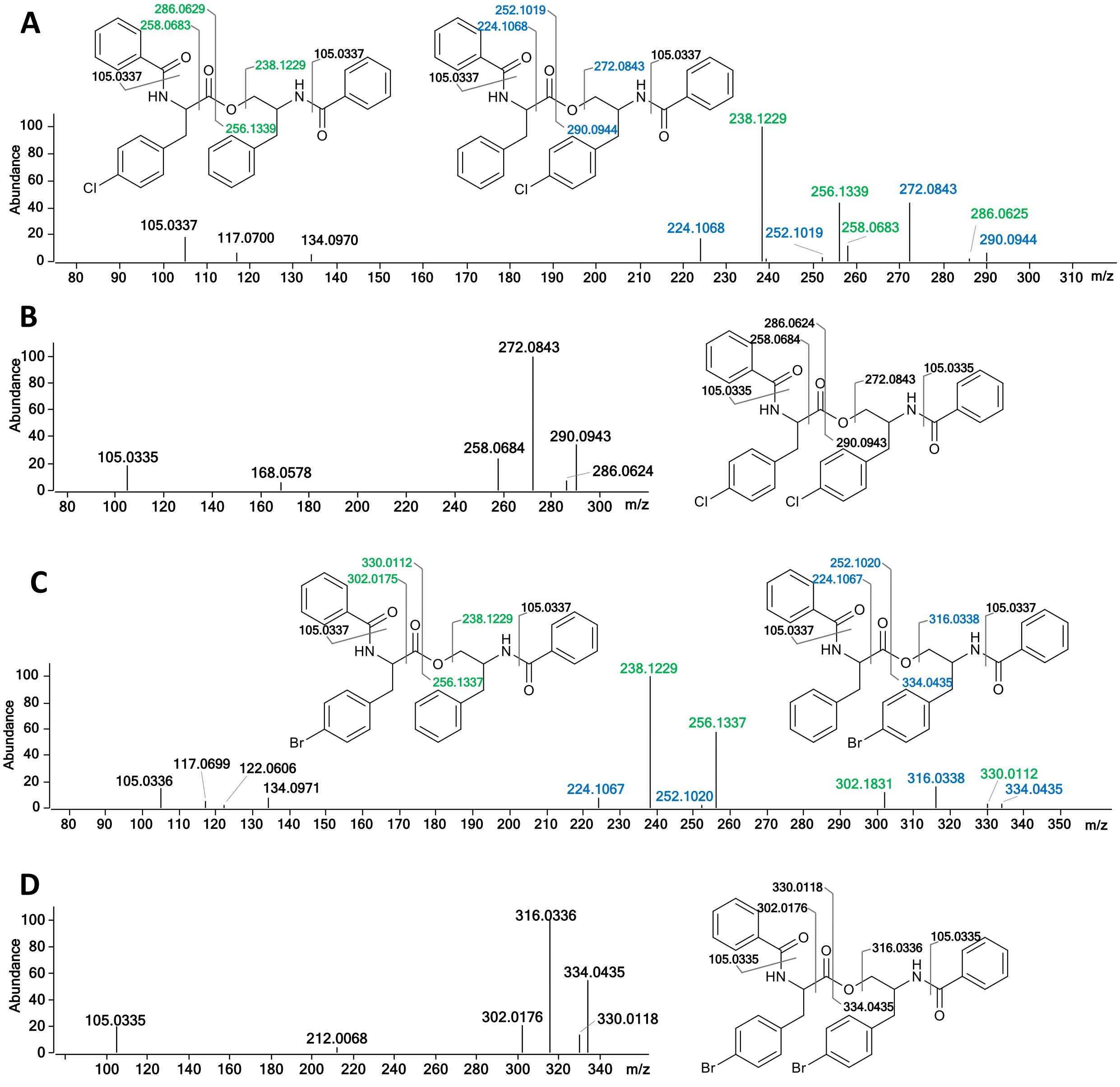

Supplementary Figure S9: MS/MS spectra and assignment of amino- and nitro- phenylalanine asperphenamate derivatives, with single substituted amino acid incorporation as well as corresponding asperphenidine analogues for p-amino-phenylalanine (AB/C) and p-nitro-phenylalanine (D/E). Fragment values highlighted in green correspond to nicotinic acid incorporation in the non-reduced amino acid moiety, whereas fragment values highlighted in blue correspond to nicotinic acid incorporation in the reduced amino acid moiety.

A

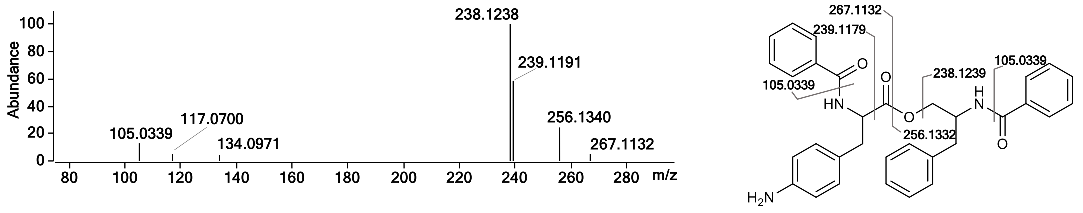

B

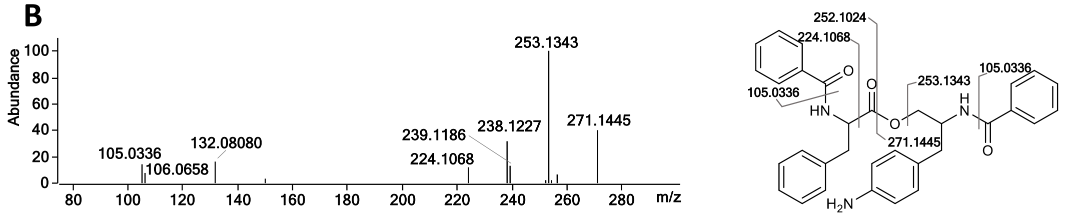

C

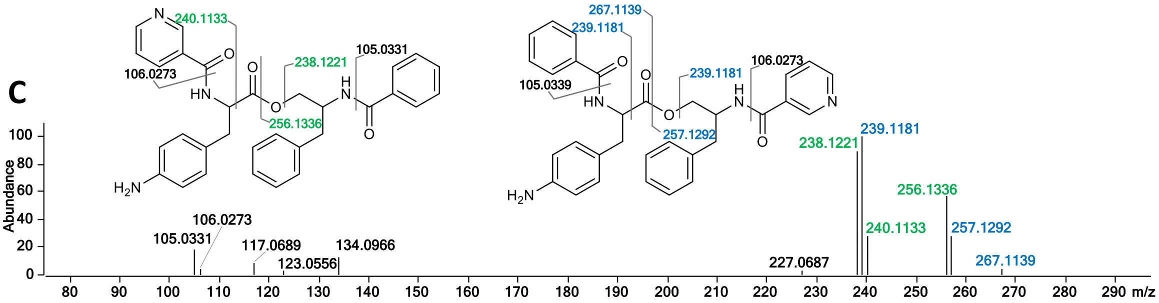

D

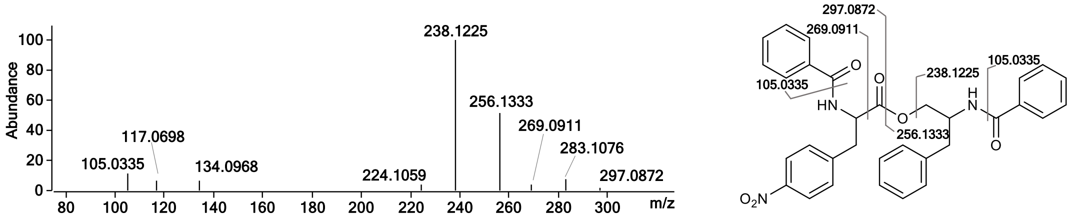

E

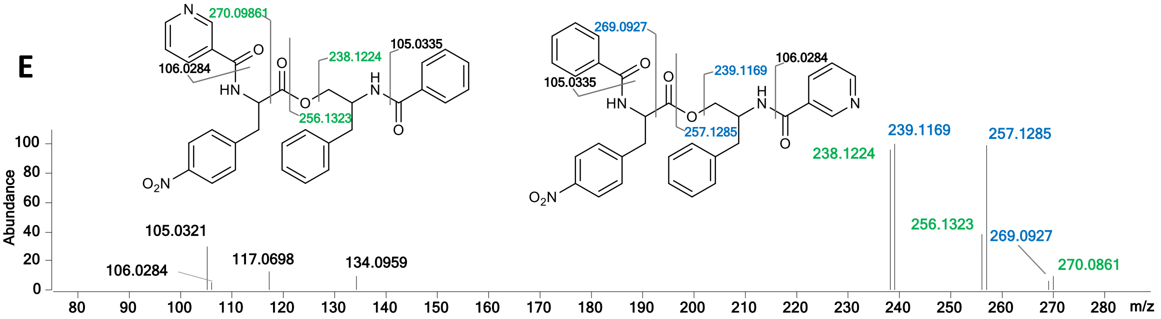

Supplementary Figure S10: BPCs of selected fungi from *Brevicompacta* section grown on YES media. Shaded peaks are EIC of $[M+H]^+$ adducts of most abundant asperphenamate analogues: m/z 508.2231 (red), m/z 523.2227 (green), m/z 546.2387 (yellow), m/z 473.2435 (blue) and m/z 507.2278 (grey). The numbers correspond to characterized asperphenamate analogues.

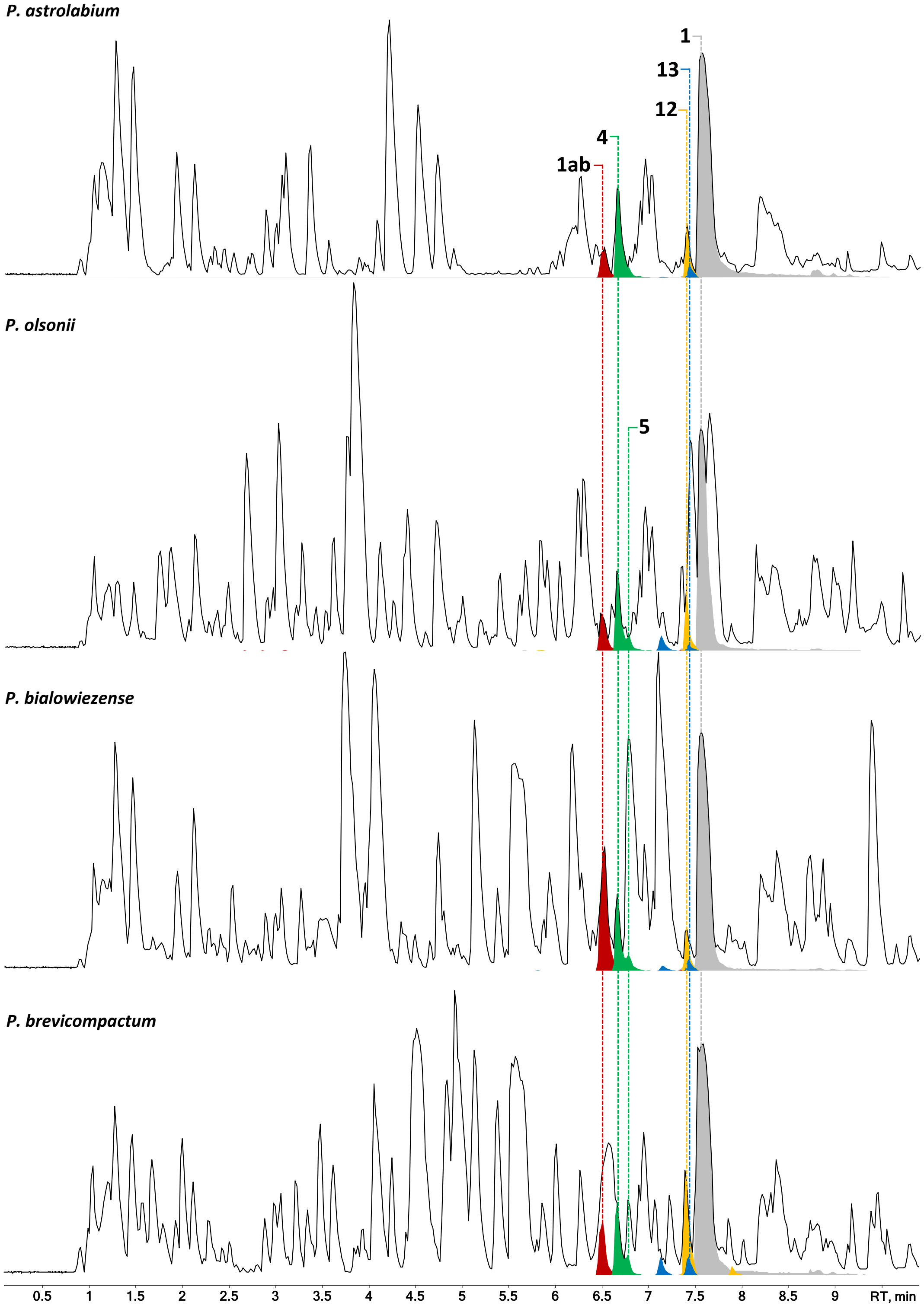

Supplement: Supplementary file 1 [file Data_Sheet_1.pdf]
